# FGF/FGFR2 Signaling Regulates the Generation and Correct Positioning of Bergmann Glia Cells in the Developing Mouse Cerebellum

**DOI:** 10.1371/journal.pone.0101124

**Published:** 2014-07-01

**Authors:** Florian Meier, Florian Giesert, Sabit Delic, Theresa Faus-Kessler, Friederike Matheus, Antonio Simeone, Sabine M. Hölter, Ralf Kühn, Daniela M. Vogt. Weisenhorn, Wolfgang Wurst, Nilima Prakash

**Affiliations:** 1 Institute of Developmental Genetics, Helmholtz Zentrum München, Deutsches Forschungszentrum für Gesundheit und Umwelt (GmbH), Neuherberg, Germany; 2 Department of Neuropathology, Regensburg University Hospital, Regensburg, Germany; 3 Centre of Genetics Engineering (CEINGE) Biotecnologie Avanzate, European School of Molecular Medicine and Institute of Genetics and Biophysics “A. Buzzati-Traverso”, Naples, Italy; 4 Technische Universität München-Weihenstephan, Lehrstuhl für Entwicklungsgenetik c/o Helmholtz Zentrum München, Neuherberg, Germany; 5 Deutsches Zentrum für Neurodegenerative Erkrankungen (DZNE) Standort München, München, Germany; 6 Max-Planck Institute of Psychiatry, München, Germany; 7 Munich Cluster for Systems Neurology (SyNergy), Adolf-Butenandt-Institut, Ludwig-Maximilians-Universität München, München, Germany; University of Nebraska Medical Center, United States of America

## Abstract

The normal cellular organization and layering of the vertebrate cerebellum is established during embryonic and early postnatal development by the interplay of a complex array of genetic and signaling pathways. Disruption of these processes and of the proper layering of the cerebellum usually leads to ataxic behaviors. Here, we analyzed the relative contribution of Fibroblast growth factor receptor 2 (FGFR2)-mediated signaling to cerebellar development in conditional *Fgfr2* single mutant mice. We show that during embryonic mouse development, *Fgfr2* expression is higher in the anterior cerebellar primordium and excluded from the proliferative ventricular neuroepithelium. Consistent with this finding, conditional *Fgfr2* single mutant mice display the most prominent defects in the anterior lobules of the adult cerebellum. In this context, FGFR2-mediated signaling is required for the proper generation of Bergmann glia cells and the correct positioning of these cells within the Purkinje cell layer, and for cell survival in the developing cerebellar primordium. Using cerebellar microexplant cultures treated with an FGFR agonist (FGF9) or antagonist (SU5402), we also show that FGF9/FGFR-mediated signaling inhibits the outward migration of radial glia and Bergmann glia precursors and cells, and might thus act as a positioning cue for these cells. Altogether, our findings reveal the specific functions of the FGFR2-mediated signaling pathway in the generation and positioning of Bergmann glia cells during cerebellar development in the mouse.

## Introduction

During vertebrate development, the cerebellum is folded into lobes and lobules with a well-defined cellular architecture comprising three cell layers, namely the outer molecular layer (ML), the Purkinje cell layer (PCL) containing Purkinje cells (PCs) and Bergmann glia (BG), and the granular layer (GL) made up of granule cells (GCs) [1], [2]. The aberrant generation during embryonic development or degeneration during adulthood of these main cerebellar layers and cell types can cause ataxic behaviors, thus underscoring the essential role of the cerebellum for motor coordination in vertebrates [3]. At around embryonic day (E) 9.0 in mice, the cerebellar anlage (CbA) is specified in the dorsal part of the anterior hindbrain under the influence of the isthmic organizer located at the boundary between the midbrain and the hindbrain [4], [5]; reviewed by [1], [2], [6]. Shortly after, at around E10–E12.5, neurons of the deep cerebellar nuclei are among the first cells generated in the CbA [7], [8]. Between E10 and E13, PCs are born in the cerebellar ventricular zone (VZ) lining the fourth ventricle, and migrate radially into the CbA along radial glia (RG) fibers spanning from the ventricular to the pial surface of the CbA [7], [9]–[12]. PCs accumulate in a multilayer underlying a second germinal zone in the outer CbA (the external granular layer (EGL)) and later form a monolayer, the PCL, in the adult cerebellar cortex. The EGL consists of granule cell precursors (GCPs) deriving from the rhombic lip at around E12 and migrating tangentially over the CbA surface until approx. E16 in mice [7], [11]. BG precursors are born in the cerebellar VZ at around E13, and migrate radially into the CbA from E14 onwards to settle among the PCs in the PCL [13]. Around birth, GCPs begin to generate postmitotic GCs that migrate along the unipolar fibers of mature BG cells past the PCs to the internal granular layer (IGL), giving rise to the GL of the adult cerebellum. The ML, containing postnatally born stellate and basket interneurons and BG fibers ensheathing the GC axons and PC dendrites, is established as the outer layer of the adult cerebellum when the inward migration of GCs has ceased [1], [2].

The Sonic hedgehog (SHH) and Fibroblast growth factor (FGF) signaling pathways play a particularly prominent role during cerebellar development. SHH secreted from PCs controls the proliferation and subsequent differentiation of GCPs [14]–[18]. The specific functions of the FGF/FGF receptor (FGFR) signaling pathway(s), by contrast, still remain unclear due to the overlapping expression domains of several FGFs and of the four known FGFRs in the developing cerebellum [19], and because of the multiple postnatal cerebellar defects in the corresponding mouse mutants [20]–[22]. Conditional ablation of the *Fgf9* or *Fgfr1*/*Fgfr2* gene(s) in neural progenitors and RG cells results in similar postnatal cerebellar phenotypes [20], [21], suggesting that FGF9 is one of the principal FGFR1/FGFR2 ligands in the developing cerebellum. In other cellular contexts, neuron-derived FGF9 binds to FGFR2 expressed on glial cells and acts as a potent survival factor [23]–[26].

We show here that the transcription of *Fgfr2* within the developing CbA initiates after E14.5 and comprises mostly cells within the anterior CbA of the developing mouse embryo. Conditional ablation of *Fgfr2* in neural progenitors results in a strong reduction of FGF signaling, in a reduced generation of BG cells and their aberrant positioning within the EGL, and in a decreased cell survival in the anterior CbA, leading to BG and PC defects already during prenatal cerebellar development. We also show that FGF9/FGFR2-mediated signaling inhibits the outward migration of RG/BG cells *in vitro*, and might thereby control their proper positioning within the PCL during cerebellar development *in vivo*.

## Materials and Methods

### Mice

Generation and genotyping of *Fgfr2^lox/lox^*, *Nestin-Cre* and *R26R* mice was described previously [27]–[29]. Mice were kept on mixed (C57BL/6J, CD-1 and 129Sv) genetic backgrounds. CD-1 mice were purchased from Charles River (Kisslegg/Germany). *Nestin-Cre* mice were mated to *Fgfr2^lox/lox^* mice and the resulting *Nestin-Cre*;*Fgfr2^+/lox^* and *Fgfr2^+/lox^* offspring was intercrossed to obtain *Nestin-Cre;Fgfr2^lox/lox^* animals, which were maintained by mating to *Fgfr2^lox/lox^* mice. Adult (2–7 months old) *Nestin-Cre;Fgfr2^lox/lox^* mice (n = 25 for histological analysis) were compared to their *Fgfr2^lox/lox^* littermate controls (n = 17 for histological analysis). For embryonic analyses, *Fgfr2^lox/lox^*;*R26R/R26R* females were mated to *Nestin-Cre*;*Fgfr2^+/lox^* males, and the *Nestin-Cre*;*Fgfr2^lox/lox^*;*R26R/+* mutant offspring (n = 28) was compared to *Nestin-Cre*;*Fgfr2^+/lox^*;*R26R/+* heterozygote controls (n = 12). The CbA phenotype of the analyzed *Nestin-Cre*;*Fgfr2^lox/lox^*;*R26R/+* mutants was routinely checked by in situ hybridization of serial sections from these embryos with a Tenascin C (*Tnc*) riboprobe. Collection of embryonic stages was done from timed-pregnant females, noon of the day of vaginal plug detection was designated as E0.5. This study was carried out in strict accordance with the recommendations in the Guide for the Care and Use of Laboratory Animals of the European Union and of the Federal Republic of Germany (TierSchG). The protocol was approved by the Institutional Animal Care and Use Committee (Ausschuss für Tierversuche und Versuchstierhaltung, ATV) of the Helmholtz Zentrum München. All efforts were made to minimize suffering.

### Behavioral tests

12 weeks old male *Nestin-Cre;Fgfr2^lox/lox^* (n = 12) and *Fgfr2^lox/lox^* control (n = 15) mice were tested in the modified hole board as described previously [30]. Motor coordination and balance was assessed one week later using the rotating rod apparatus (Rotarod Letica LE 8200, Bioseb/France). The test phase consisted of three trials separated by 15 min intertrial intervals. Per trial, three mice were placed on the rod leaving an empty lane between two mice. The rod was initially rotating at 4 rpm constant speed to allow positioning of all mice in their respective lanes. Once all mice were positioned, the rod accelerated from 4 to 40 rpm in 300 s, and passive rotations or latency and rpm at which each mouse fell off the rod were recorded.

### EdU treatments

Pregnant dams were injected intraperitoneally with 10 µg 5-ethynyl-2′-deoxyuridine (EdU; Invitrogen/Germany) per gram body weight on E17.5. Embryos were dissected 24 h later and processed for EdU detection on paraffin sections according to the manufacturer's instructions (Click-iT EdU Alexa Fluor 488 Imaging Kit, Invitrogen).

### Radioactive in situ hybridization (ISH)

Serial paraffin sections (8 µm) from embryonic and adult mouse heads or brains were hybridized with radioactive ([α-^35^S]UTP, GE Healthcare/USA) or Digoxigenin (DIG RNA Labeling Mix, Roche/Germany) labeled riboprobes as described previously [31]–[33]. Riboprobes used were *Fgfr2 exon 5* and *Etv5* (*Erm*) [27], *Gad2* (*GAD65*) and *VAChT* (*Slc18a3*) [34], *Fgfr1*, *Fgfr2*, *Fgfr3* and *Fgfr4* [35], Tenascin C (*Tnc*) [36], *Atoh1* (*Math1*) [37], *Th* and *Sert* (*Slc6a4*) [31], *Shh* [38], and Patched 1 (*Ptch1*) [39]. Images were taken with an Axioplan2 microscope or StemiSV6 stereomicroscope using bright- and darkfield optics, AxioCam MRc camera and Axiovision 4.6 software (Zeiss/Germany), and processed with Adobe Photoshop CS5 software (Adobe Systems Inc./USA).

### Immunohisto-/cytochemistry (IHC/ICC)

Antigens were detected on paraffin sections (8 µm), free-floating cryosections (40 µm), or microexplant cultures as reported by [31], [40], [41]; minor modifications are available upon request. Primary antibodies used were mouse anti-Gfap (glial fibrillary acidic protein) (1∶1000; Sigma/Germany), anti-Calb1 (calbindin) (1∶200; Swant/Switzerland, CB300), anti-Blbp (Brain lipid binding protein, Fabp7) (1∶300; Millipore/USA) and anti-Pax6 (1∶400; Developmental Studies Hybridoma Bank/USA); rabbit anti-Calb2 (calretinin) (1∶2000; Swant), anti-S100b (1∶1000; Sigma), anti-Calb1 (1∶5000 (embryos), 1∶2000 (adult tissues); Swant, CB38a), anti-Ccnd1 (Cyclin D1) (1∶150; Thermo Fisher Scientific/USA), anti-Glast (Glial high affinity glutamate transporter, Slc1a3, Eaat1) (1∶100; Abcam/UK), anti-phosphorylated Histone H3 (pH3) (1∶1000; Millipore) and anti-cleaved (activated) Caspase 3 (cCasp3) (1∶150; Cell Signaling Technologies/USA); and goat anti-Sox2 (1∶500; Santa Cruz Biotechnology/USA). Secondary antibodies were either fluorescently labeled conjugates (AlexaFluor 488/546/594, 1∶500; Invitrogen) counterstained with 4′,6-diamidino-2-phenylindole (DAPI), or coupled to horseradish peroxidase and detected using the Vectastain ABC Elite Kit (Vector Laboratories/USA). Images were taken with an Axiovert 200M or Axioplan 2 microscope and AxioCam HRc or MRc camera (Zeiss), or with an Olympus IX81 confocal laser scanning microscope (Olympus/Germany), and processed with Adobe Photoshop CS5 software.

### Cell countings

Cells were counted in an area corresponding to approx. the anterior part (anterobasal lobe) of the CbA on 5 to 8 midsagittal sections from E16.5 and E18.5 control and *Nestin-Cre*;*Fgfr2^lox/lox^*;*R26R/+* embryos using Stereo Investigator 5.05.4 software (MBF Bioscience/USA). *Tnc* (*Tnc* ^+^) and cCasp3 (cCasp3^+^) -expressing cells were normalized to this area (except the EGL area for cCasp3^+^ cells); Pax6 (Pax6^+^) and EdU (EdU*^+^*) -expressing cells were normalized to the Pax6^+^ EGL area; and pH3 (pH3^+^) -expressing cells were normalized to the VZ area of the cerebellum.

### Western blot

Brain tissues were isolated from 8 weeks old *Fgfr2^lox/lox^* (control), heterozyote *Nestin-Cre*;*Fgfr2^+/lox^* and homozygote *Nestin-Cre*;*Fgfr2^lox/lox^* mice, and homogenized in RIPA buffer (50 mM Tris-HCl pH 7.4, 150 mM NaCl, 1% NP-40, 0.25% Sodium deoxycholate, 1 mM EDTA, and Complete protease inhibitors (Roche)). Total protein concentration was determined with the Pierce BCA Protein Assay (Thermo Fisher Scientific), and 50 µg total protein per sample were separated in 10% NuPAGE Novex precast gels (Invitrogen) and blotted onto PVDF membranes (Hall/USA). Blots were blocked in 4% skim milk in TBST (50 mM Tris-HCl pH 7.5, 150 mM NaCl, 0.05% Tween 20) and probed with rabbit anti-Fgfr2 (1∶300; sc-122) and anti-hypoxanthine guanine phosphoribosyl transferase (Hprt) (1∶400; FL-218) antibodies (both from Santa Cruz Biotechnology). Membranes were developed in ECL substrate and exposed to Hyperfilm ECL (GE Healthcare).

### CbA microexplant cultures

CbA microexplant cultures were prepared essentially as described by Kunemund et al. (1988) [42], with some modifications to account for the embryonic tissues and the smaller size of the CbA. Briefly, cerebellar primordia were isolated from E16.5 CD-1 embryos, cut into small pieces of equal size (approx. 750 µm diameter), and plated onto poly-D-lysine (50 µg/ml; Millipore) and laminin (2 µg/ml; Roche) coated coverslips (1 microexplant/coverslip) in Neurobasal medium supplemented with 2 mM L-glutamine, 1x B27 nutrient mixture, 100 Units/ml penicillin, 100 µg/ml streptomycin (all from Invitrogen), and 200 nM ascorbic acid (Sigma). Immediately after plating, 100 ng/ml recombinant human FGF9 protein (R&D Systems/USA) in bovine serum albumin (BSA), 20 µM InSolution SU5402 (Merck Chemicals/Germany) in dimethyl sulfoxide (DMSO) or 0.0001% BSA and 0.1% DMSO (control) were added to the medium. Microexplants were incubated at 37°C in a humidified 5% CO_2_ atmosphere for 36 h, and then fixed in 4% paraformaldehyde, processed for Ccnd1 and Pax6 ICC as described before and counterstained with DAPI.

### Migration assays

To measure the outward migration of cells from the core of the CbA microexplants, we employed a method that was originally described by Chou et al. (2000) [43]. Briefly, the border of the microexplant was outlined and the average distance migrated by Pax6^−^/Ccnd1^+^ (RG/BG), Pax6^+^/Ccnd1^+^ (GCP) and Pax6^+^/Ccnd1^−^ (GC) cells (nuclei) from the border on one side of the microexplant was measured using the Neurolucida 6 and Neurolucida Explorer software (MBF Bioscience). The distribution of migrating cells was categorized in 50-µm bins (distance migrated from the border of the microexplant) using the following procedure: a computer-generated 50×50 µm grid was superimposed on the image on the side of the microexplant where cells had migrated out. The number of Pax6^−^/Ccnd1^+^ (RG/BG), Pax6^+^/Ccnd1^+^ (GCP) and Pax6^+^/Ccnd1^−^ (GC) cells (nuclei) within the 0–50, 50–100, 100–150, 150–200, 200–250, 250–300, 300–350 and 350–400 µm bins was counted, and the correct assignment of the cells to each of these bins was inspected using the Neurolucida Explorer software. A minimum of six microexplants were analyzed for each condition, and data are derived from three independent experiments.

### Statistical analyses

All values given are mean ± s.e.m., unless otherwise indicated. Statistical analysis was performed with the SPSS v.10.0 (SPSS Inc., Chicago/USA) and GraphPad Prism 6 (GraphPad Inc./USA) software for the behavioral data, and R software [44] for the cell counting and cell migration data. Modified hole board, rotating rod, cell counts and average migrated distance data were analyzed by one-way or two-way ANOVA, and *P*<0.05 was considered as significant. Students *t*-tests were used for post-hoc comparisons when appropriate. Mann-Whitney U-tests were used for analysis of rearing behavior data. In case of a non-significant interaction, *P*-values were taken for genotype or treatment factor. For the cell migration data, the proportion of Pax6^−^/Ccnd1^+^ RG/BG precursors/cells among all Ccnd1^+^ and Pax6^+^ cells was analyzed with a logistic model for the influence of treatments, considering the grouping of the data according to different experiments (generalized linear mixed-effects model); the R package lme4 [45] was used for these calculations. Confidence intervals for the proportions were calculated with a random intercept-only model.

## Results

### *Fgfr2* deficiency leads to locomotor deficits and an aberrant cerebellar organization in adult mice

**Figure 1:**
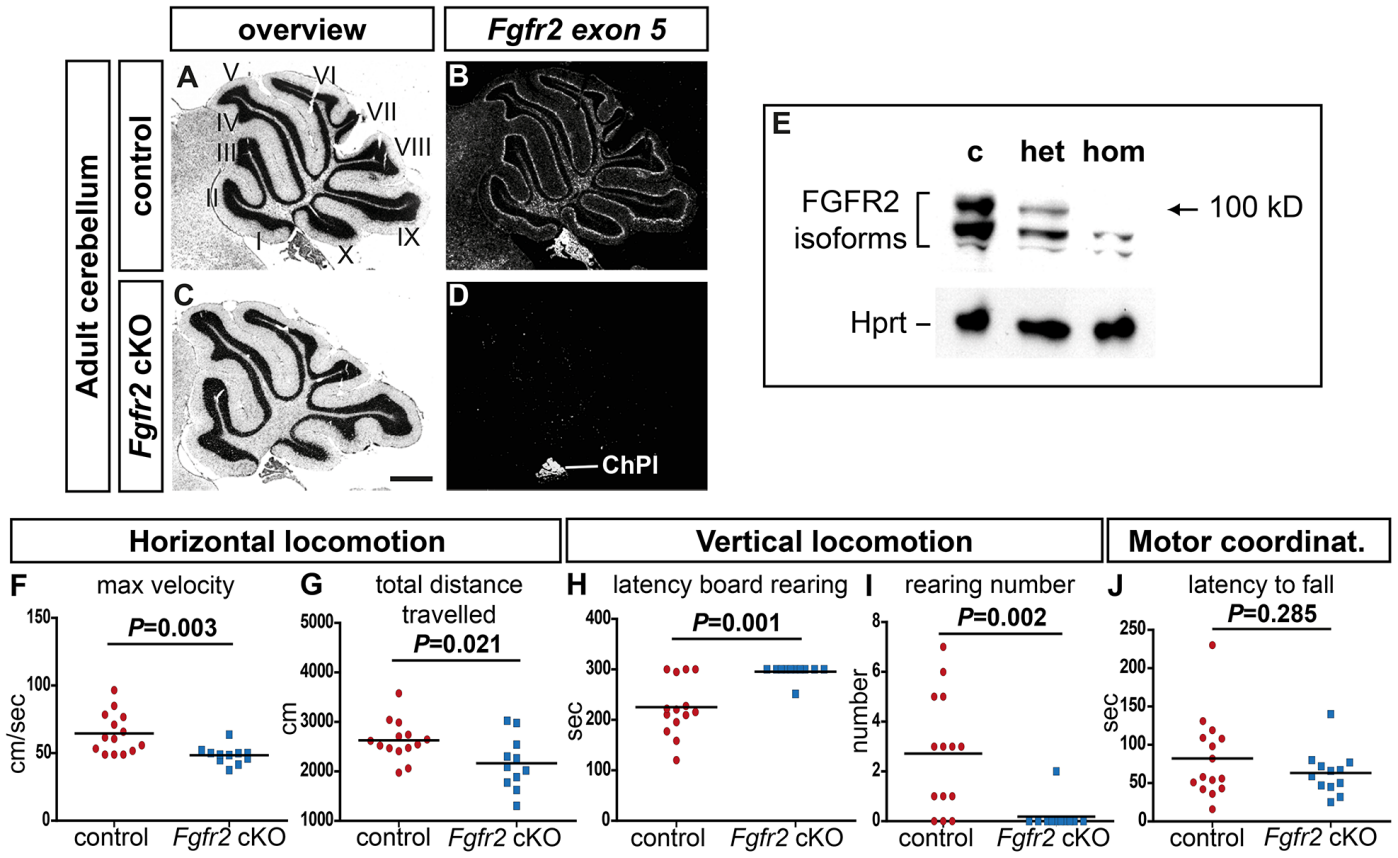
*Fgfr2* deficiency leads to locomotor deficits in adult mice. (**A–D**) Cresyl-violet stained brightfield (A,C) and darkfield (B,D) views of sagittal sections from adult *Fgfr2^lox/lox^* (control, A,B) and *Nestin-Cre;Fgfr2^lox/lox^* (*Fgfr2* cKO, C,D) cerebella, hybridized with a radioactive *Fgfr2 exon 5* riboprobe. (**E**) Western blotting detected the full-length FGFR2 protein (approx. 100 kD) in brain lysates of adult *Fgfr2^lox/lox^* (c, control) and *Nestin-Cre;Fgfr2^+/lox^* (het, heterozygote) but not *Nestin-Cre;Fgfr2^lox/lox^* (hom, homozygote) mice. Hprt is the loading control. (**F–J**) Behavioral tests revealed an altered horizontal locomotion (maximum velocity (F) and total distance travelled (G)) and unsupported vertical locomotion (latency to first rearing (H) and number of rearings (I) on the board) of male *Fgfr2* cKO (blue squares; n = 12 males) compared with control (red circles; n = 15 males) mice in the modified hole board paradigm, but no significant differences between both genotypes in the accelerating Rotarod performance (measured by mean latency to fall, J). Values are given in Table S1. I-X, lobuli of the adult cerebellum; ChPl, choroid plexus. Scale bar (C): 500 µm.

To establish the function of FGFR2 in developing neural progenitors, we generated *Nestin-Cre;Fgfr2^lox/lox^* conditional knock-out (henceforth designated as *Fgfr2* cKO) mice, in which deletion of exon 5 leads to a premature stop codon in exon 6 and truncation of the FGFR2 protein at the extracellular Ig-like II domain [27]. *Fgfr2 exon 5*-specific mRNA was only detected in the choroid plexus (ChPl) but not in the cerebellum of adult (>8 weeks old) *Fgfr2* cKO mice (Figure 1A–D), and full-length FGFR2 protein was not detectable in the brains of these mice using an antibody raised against the C-terminus of FGFR2 (Figure 1E). Adult *Fgfr2* cKO mice were viable and fertile; to assess their locomotor abilities, we tested these mice on the modified hole board (mHB) [30] and rotating rod (Rotarod). In the mHB test, *Fgfr2* cKO males showed a significantly decreased maximum velocity and reduced total distance travelled (Figure 1F,G; Table S1 in File S1), indicating an altered horizontal locomotion. The mutant males also showed a significantly increased latency to the first rearing and reduced rearing frequency on board in the middle of the arena (Figure 1H,I; Table S1 in File S1), indicating an altered vertical locomotion when this was not supported by the arena wall. However, the Rotarod performance (latency to fall) of the *Fgfr2* cKO males was not significantly different from control males (Figure 1J; Table S1 in File S1), although we noted some variation in the latencies to fall from the Rotarod among the *Fgfr2* cKO males (Figure S1 in File S1). Because of these results, we focused our further analyses on the cerebellum of the *Fgfr2* cKO mice, although we cannot exclude that the loss of FGFR2 in other brain regions of these mice also contributed to the observed locomotor phenotype. Nevertheless, we did not observe any obvious alterations in the ventral mid-/hindbrain region (MHR) of the *Fgfr2* cKO mice, and dopaminergic, noradrenergic, serotonergic and cholinergic neurons located in this region appeared unaffected by the ablation of *Fgfr2* in their progenitors (Figure S2 in File S1).

**Figure 2:**
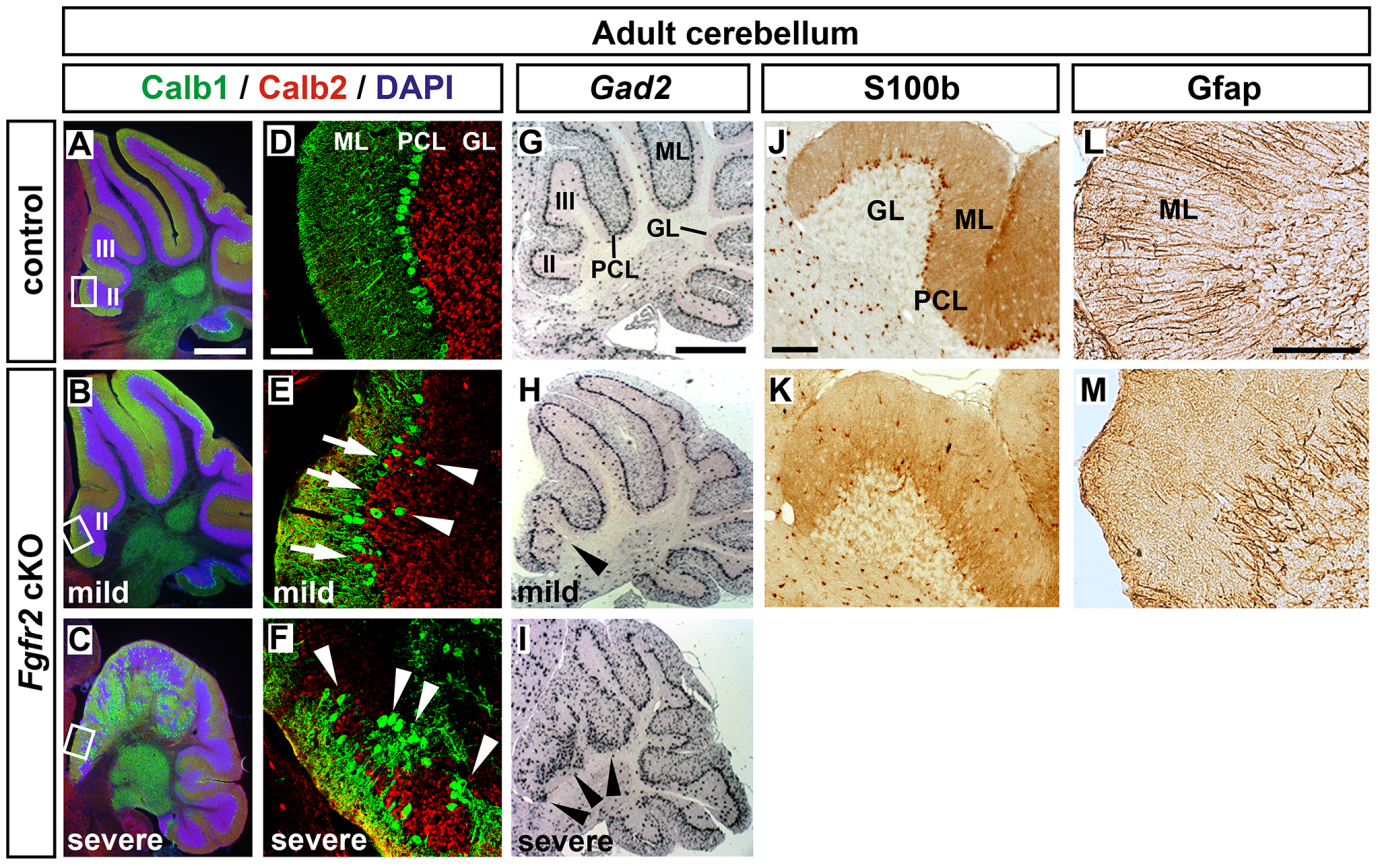
Aberrant cellular layering and organization of the adult *Fgfr2* cKO cerebellum. (**A–F**) Representative confocal images of sagittal sections from adult control (A; n = 2 mice) and *Fgfr2* cKO (B,C; n = 5 mice) cerebella, double-immunostained for calbindin (Calb1, green) and calretinin (Calb2, red), and counterstained with DAPI (blue). (D–F) are higher magnifications of the boxed areas in (A–C). White arrowheads in (E) point at ectopically located Calb1^+^ PCs within the GL, and white arrows in (E) denote the PC “gaps” within the PCL occupied by Calb2^+^ GCs. White arrowheads in (F) point at Calb1^+^ PC clusters surrounded by Calb2^+^ GCs. (**G–I**) Representative sagittal sections from adult control (G; n = 15 mice) and *Fgfr2* cKO (H,I; n = 20 mice) cerebella hybridized with a riboprobe for *Gad2*, which is expressed in PCs and GABAergic interneurons within the ML. Black arrowheads in (H,I) point at the disorganized *Gad2* ^+^ PCL in the anterior lobules (II and III) of the mutant cerebella. The cerebella shown in (B,E,H) display a milder phenotype, which was detected in 8/25 (32%) of the *Fgfr2* cKO mice. The cerebella shown in (C,F,I) display a more severe phenotype, detected in 15/25 (60%) of the mutants. (**J–M**) Representative immunostainings for S100b (J,K) and Gfap (L,M) on sagittal sections from adult control (J,L) and less severely affected *Fgfr2* cKO (K,M) cerebella. Note the reduced numbers of S100b^+^ BG cells within the PCL and ectopic positioning of S100b^+^ BG cells within the ML in the mutant cerebellum. Gfap^+^ BG fibers did not reach the pial surface of the mutant cerebellum. GL, granular layer; ML, molecular layer; PCL, Purkinje cell layer; II and III, lobuli of the adult cerebellum. Scale bars: 500 µm (A,G), 50 µm (D), 100 µm (J,L).

A detailed histological analysis of 25 adult *Fgfr2* cKO cerebella revealed that the cellular architecture of the cerebellum, particularly in the anterior lobules (lobuli II and III), was severely disrupted in 15 (60%) mutants (Figure 2C,F,I) and was less severely affected in 8 (32%) of the mutant mice (Figure 2B,E,H). In the less severely affected mutant cerebella, Calbindin-expressing (Calb1^+^) PCs were misaligned in the presumptive PCL and some PCs were located within the GL, whereas Calretinin-expressing (Calb2^+^) GCs intermingled with the Calb1^+^ PCs and occupied the gaps devoid of PCs in the PCL (Figure 2E). By contrast, the PCL and GL were completely disrupted in the severely affected *Fgfr2* cKO cerebella, and clusters of Calb1^+^ PCs were surrounded by Calb2^+^ GCs in these mice (Figure 2F). Because of these strong morphological and cellular alterations in the severely affected *Fgfr2* cKO cerebella, we subsequently analyzed only the less severely affected specimens. In these mutant mice, the numbers of S100b^+^ BG cells appeared to be reduced in the anterior lobuli, several S100b^+^ BG cell bodies were ectopically located in the ML, and the Gfap^+^ radial fibers of the BG cells did not reach the pial surface of the adult *Fgfr2* cKO cerebellum (Figure 2J–M). We thus concluded that the inactivation of *Fgfr2* in neural progenitors of the developing mouse cerebellum leads to locomotor deficits and the disruption of the normal cellular organization and layering of the adult cerebellum at a variable penetrance. Because a similar albeit stronger postnatal cerebellar phenotype has been reported by Lin et al. (2009) [20] in *Fgfr1*/*Fgfr2* double mutant mice generated with the same conditional mutagenesis approach, we focused our subsequent analyses on the relative contribution of FGFR2 signaling to the developmental defects underlying these phenotypes.

### Localized expression of *Fgfr2* in the developing mouse CbA

**Figure 3:**
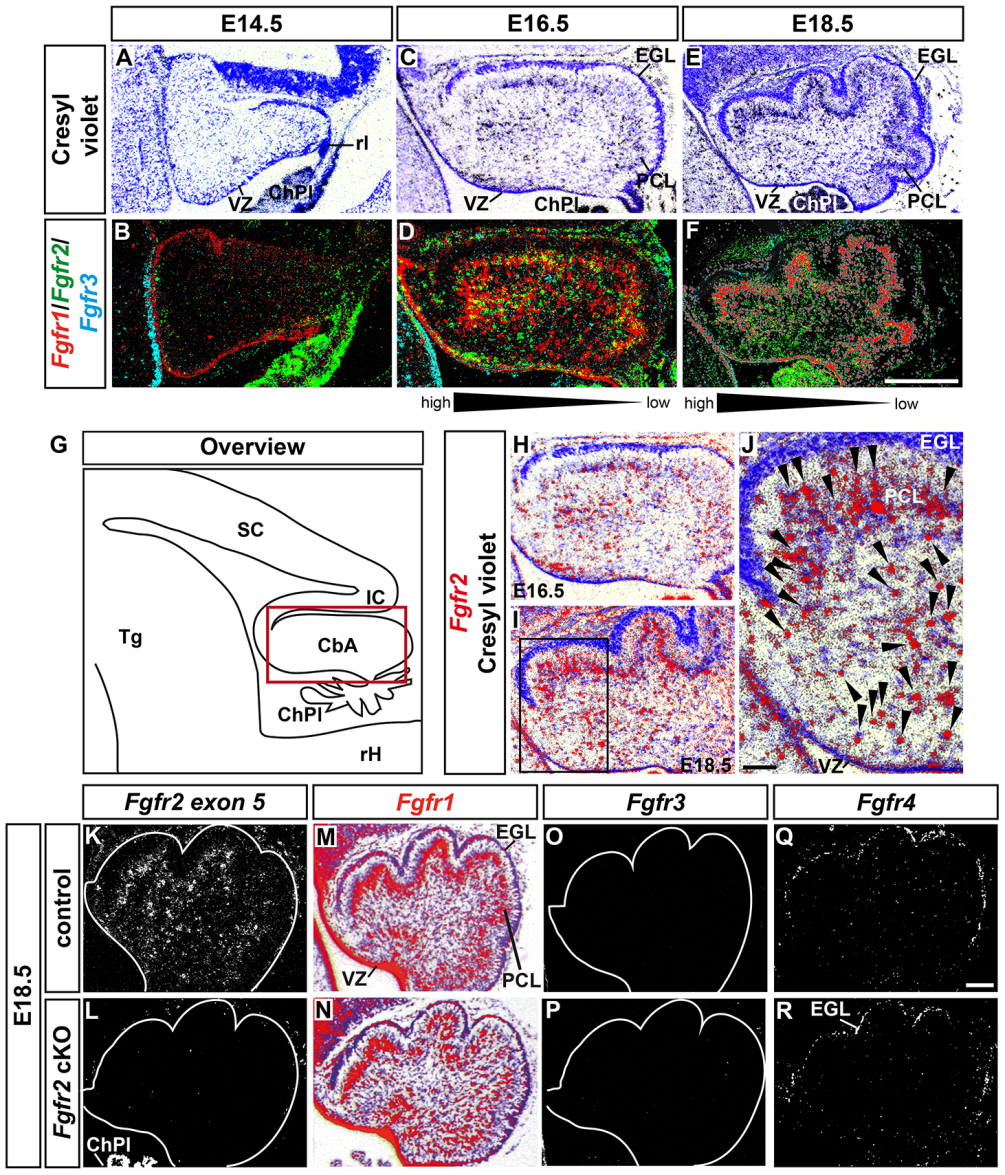
Expression of *Fgfr2* within the developing mouse CbA. (**A–F**) Representative brightfield views of Cresyl-violet-stained midsagittal sections (A,C,E) and darkfield views of pseudo-colored overlays from consecutive midsagittal sections hybridized with radioactive *Fgfr1* (red), *Fgfr2* (green) and *Fgfr3* (blue) riboprobes (overlapping expression domains appear in yellow) (B,D,F) through the CbA of wild-type (CD-1) mouse embryos at E14.5 (A,B; n = 5 embryos), E16.5 (C,D; n = 5 embryos) and E18.5 (E,F; n = 6 embryos). (**G**) Schematic overview of a midsagittal section through the embryonic MHR. The red box delimits the CbA, shown at higher magnification in (H,I). (**H–J**) Cresyl-violet-stained and pseudo-colored midsagittal sections through the CbA of wild-type (CD-1) mouse embryos at E16.5 (H, same section as in C) and E18.5 (I,J, same section as in E) hybridized with a radioactive *Fgfr2* riboprobe (red). (J) is a higher magnification of the boxed area in (I). Note that the majority of the *Fgfr2*-expressing cells are intensely Nissl-stained (glial) cells (black arrowheads in J) located mostly in the anterior CbA at these stages (depicted by the black triangle and high→low gradient below (D,F)). (**K–R**) Representative midsagittal darkfield (K,L,O-R) and brightfield (M,N) views of the CbA in E18.5 control (K,M,O,Q; n = 5 embryos) and *Fgfr2* cKO (L,N,P,R; n = 5 embryos) embryos hybridized with radioactive *Fgfr2 exon 5* (K,L), *Fgfr1* (M,N), *Fgfr3* (O,P) and *Fgfr4* (Q,R) riboprobes. CbA, cerebellar anlage; ChPl, choroid plexus; EGL, external granular layer; IC, inferior colliculus; PCL, Purkinje cell layer; rH, rostral hindbrain; rl, rhombic lip; SC, superior colliculus; Tg, tegmentum; VZ, cerebellar ventricular zone. Scale bars: 500 µm (F), 50 µm (J), 100 µm (Q).

Using a sensitive radioactive ISH method, we first assessed the wild-type (CD-1) expression pattern of *Fgfr2* in relation to *Fgfr1* and *Fgfr3*, two other FGFRs expressed during prenatal development (E14.5–18.5) in the murine MHR (Figure 3A–F; [19]). *Fgfr2* is transcribed strongly in the ChPl and weakly in the VZ of the dorsal midbrain (tectum) and in the overlying mesenchyme, but not within the CbA of the E14.5 mouse embryo (Figure 3A,B; Figure S3 in File S1). At E16.5, *Fgfr2* is expressed in single cells that appear to delaminate from the cerebellar VZ and to migrate towards the cerebellar cortex, because several *Fgfr2*-expressing cells have already accumulated in the prospective PCL (Figure 3C,D,H). At this stage, *Fgfr2* is also expressed strongly in the ChPl and in the VZ of the dorsal midbrain (comprising the superior and inferior colliculi), and weakly in the overlying mesenchyme of the MHR (Figure S3 in File S1). At E18.5, strongest expression of *Fgfr2* is still detected in single cells located within the CbA that appear to migrate towards the cerebellar cortex and to assemble within the forming PCL (Figure 3E,F,I,J). *Fgfr2*-expressing cells are not detected in the EGL, and only few *Fgfr2* ^+^ cells are located between the prospective PCL and EGL (the prospective ML) at this stage (Figure 3I,J). Because we detected the majority of the *Fgfr2*-expressing cells in the anterior CbA at E16.5 and E18.5, whereas relatively fewer *Fgfr2* ^+^ cells were detected in the posterior CbA (Figure 3C–I), the expression of *Fgfr2* within the developing CbA has the appearance of a “graded” anterior^high^–posterior^low^ pattern at these stages (Figure 3D,F). At E18.5, transcription of *Fgfr2* is still strong in the ChPl but weaker in the overlying mesenchyme (Figure S3 in File S1). Throughout all analyzed stages, *Fgfr2* is not detected in the cerebellar VZ or EGL (Figure 3A–J; Figure S3 in File S1). Notably, visualization at high magnification showed that the *Fgfr2* ISH signal appeared to co-localize preferentially with intensely Nissl-stained cells at E16.5 and E18.5 (Figure 3G-J), suggesting that this receptor is expressed mainly in glial cells (RG and/or BG precursors and cells) of the CbA at these prenatal stages. In contrast to *Fgfr2*, *Fgfr1* is transcribed strongly in the VZ of the CbA from E14.5 to E18.5 (Figure 3B,D,F; Figure S3 in File S1; [19]). From E16.5 on, *Fgfr1* is also expressed in single cells that appear to migrate within the CbA and in cells that have accumulated in the emerging PCL (Figure 3D; Figure S3 in File S1; [19]). *Fgfr1* expression becomes most prominent within the PCL at E18.5, although it is still expressed in single cells within the CbA that appear to migrate towards the PCL (Figure 3F; [19]). *Fgfr1* is also transcribed in the VZ of the ventral and dorsal midbrain and rostral hindbrain throughout these stages (Figure S3 in File S1). *Fgfr3* expression is not detected in the CbA from E14.5 to E18.5 (Figure 3B,D,F), but *Fgfr3* is transcribed strongly in the VZ and in scattered cells of the rostral hindbrain, sparing the isthmic region, at these stages (Figure S3 in File S1). We thus concluded that *Fgfr2* starts to be transcribed after E14.5 in the developing CbA, and that *Fgfr2* is expressed in single cells located mostly within the anterior CbA. These *Fgfr2*-expressing cells appear to delaminate from the cerebellar VZ and to migrate in direction of the emerging PCL, where they assemble toward the end of the prenatal period. Moreover and based on their Nissl staining, these cells appear to have a glial identity. The partial overlap of *Fgfr2* and *Fgfr1* expression in the developing CbA at E16.5–18.5 (Figure 3D,F) suggests some functional redundancy between these two FGFRs in cerebellar development. However and in contrast to *Fgfr2*, *Fgfr1* is prominently expressed in the cerebellar VZ, suggesting that *Fgfr1* might additionally be involved in the generation and/or maintenance of VZ progenitor cells.

Next, we analyzed the expression of *Fgfr2* and of the other three mouse *Fgfr* genes in the CbA of the *Fgfr2* cKO embryos at E18.5 (Figure 3K–R). Transcription of *Fgfr2* was completely lost (Figure 3K,L) and *Fgfr1* appeared to be reduced in the PCL but not in the VZ of the mutant CbA (Figure 3M,N), whereas the expression of *Fgfr3* and *Fgfr4* was not altered in the mutants (Figure 3O–R), indicating that the inactivation of *Fgfr2* might have affected the expression of *Fgfr1* but not of the other two *Fgfr*s in the developing mouse cerebellum.

### Reduced numbers and mispositioning of BG cells in the EGL are the primary cerebellar defects in the *Fgfr2* cKO embryos

**Figure 4:**
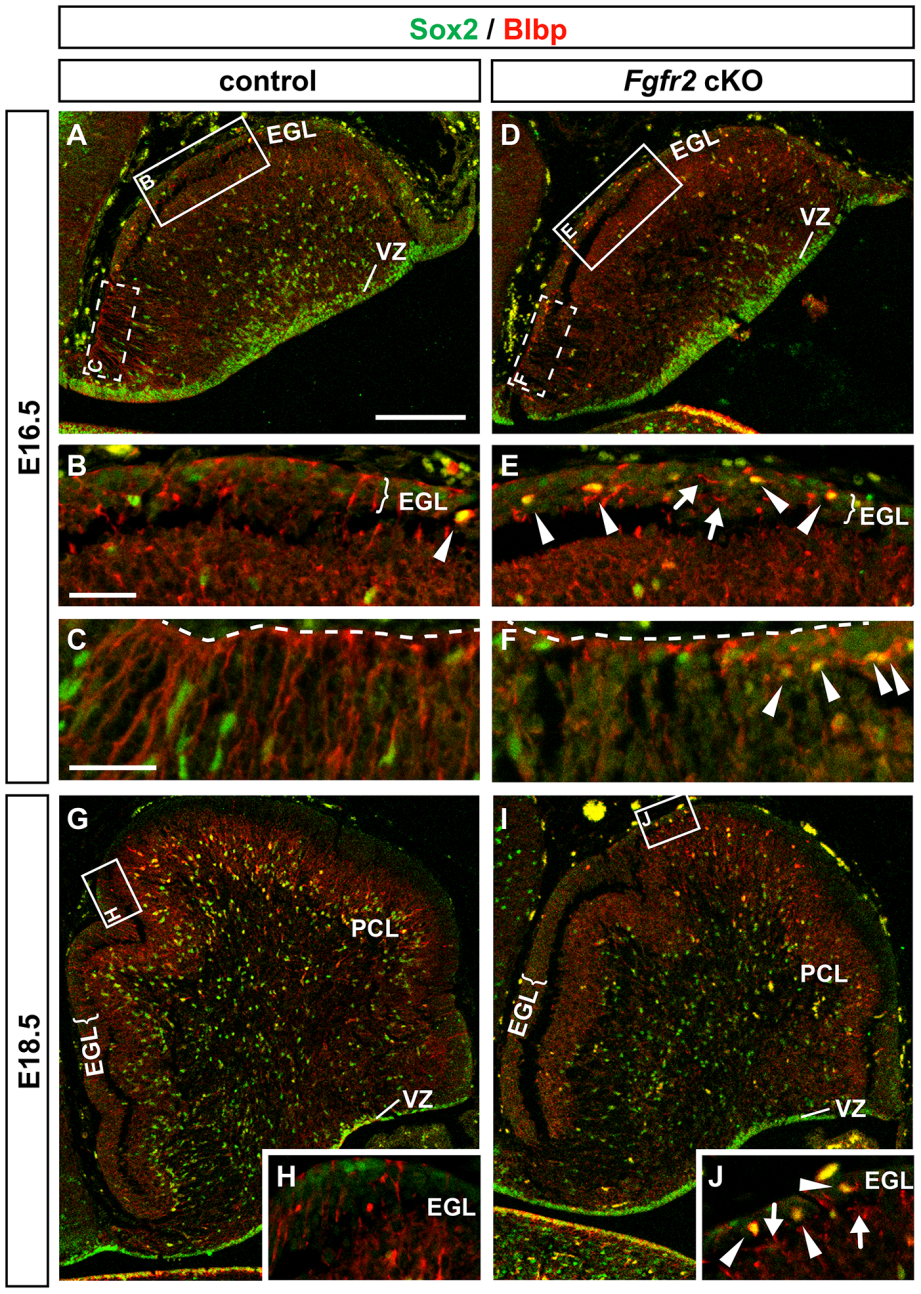
Strong reduction and ectopic positioning of Sox2^+^/Blbp^+^ BG precursors and cells in the *Fgfr2* cKO CbA. (**A–J**) Representative confocal overviews (A,D,G,I) and close-up views (B,C,E,F,H,J) of the CbA on sagittal sections from control (A,B,C,G,H) and *Fgfr2* cKO (D,E,F,I,J) embryos at E16.5 (A–F; n = 4 embryos/genotype) and E18.5 (G–J; n = 3 embryos/genotype), immunostained for Sox2 (green) and Blbp (Brain lipid binding protein, red). A marked reduction of Sox2^+^/Blbp^+^ BG precursors/cells was observed in the *Fgfr2* cKO (D,I) compared to control (A,G) embryos at both stages. A reduction of Sox2^+^ and Blbp^+^ neural progenitors and RG/BG precursors was also apparent in the anterior (left) part of the cerebellar VZ of the mutant embryos at E16.5 (A,D), whereas at E18.5, Sox2^+^ and Blbp^−^ neural progenitor cells appeared to accumulate in the cerebellar VZ of the *Fgfr2* cKO embryos (G,I). Close-up views (B,C,E,F,H,J) of the boxed areas in (A,D,G,I) revealed an increased number of ectopically positioned Sox2^+^/Blbp^+^ BG cells (white arrowheads) within the EGL of the *Fgfr2* cKO embryos at E16.5 and E18.5. Only few Blbp^+^ BG fibers reached the pial surface (dashed line in C,F) of the mutant CbA, and these fibers were frequently arranged in a parallel (tangential) rather than perpendicular (radial) manner relative to this surface (white arrows in E,J). EGL, external granular layer; PCL, Purkinje cell layer; VZ, cerebellar ventricular zone. Scale bars: 500 µm (A), 100 µm (B,C).

**Figure 5:**
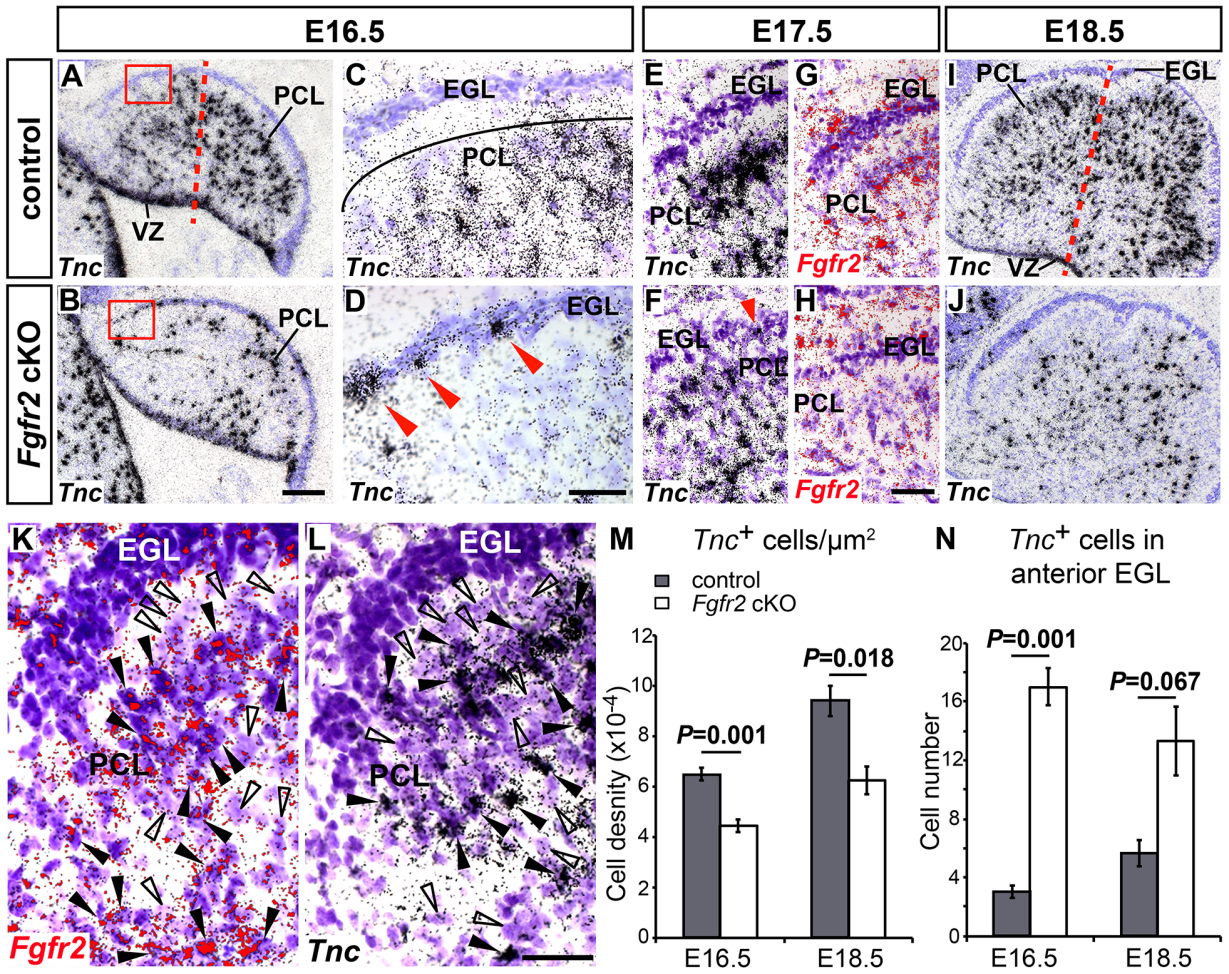
Reduced numbers and mispositioning of *Tnc* ^+^ BG cells in the EGL of the *Fgfr2* cKO CbA. (**A–J**) Representative sagittal brightfield views of E16.5 (A–D, n = 4 embryos/genotype), E17.5 (E–H, n = 1 embryo/genotype) and E18.5 (I,J, n = 3 embryos/genotype) control (A,C,E,G,I) and *Fgfr2* cKO (B,D,F,H,J) cerebella hybridized with radioactive *Tnc* (A–F,I,J) and *Fgfr2* (G,H) riboprobes. (C,D) are higher magnifications of the boxed areas in (A,B). (E–H) are higher magnifications of the anterior CbA in adjacent sections from control or mutant embryos. Red arrowheads in (D,F) point at ectopically positioned *Tnc* ^+^ BG cells in the mutant EGL. Note the complete absence of the *Fgfr2* ISH signal correlating with less *Tnc* ^+^ and intensely Nissl-stained cells in the CbA of the *Fgfr2* cKO embryo shown in (F,H), although some *Fgfr2* ^+^ cells are detected in the (non-neural) mesenchyme overlying the mutant EGL. Red dotted line in (A,I) delimits the anterior area used for quantification. (**K,L**) High magnification views of the EGL and PCL on adjacent sections from an E18.5 control (wild-type) embryo, hybridized with a radioactive riboprobe for *Fgfr2* (red in K) or *Tnc* (black in L). Black arrowheads point at intensely Nissl-stained cells showing an ISH signal for *Fgfr2* (K) or *Tnc* (L). Empty arrowheads point at larger, weakly Nissl-stained cells devoid of *Fgfr2* (K) or *Tnc* (L) ISH signals. (**M,N**) Quantification of *Tnc* ^+^ cells in the anterior CbA (M) and EGL (N) of control (grey bars) and mutant (white bars) embryos at E16.5 and E18.5 (*Tnc* ^+^ cells/µm^2^ (M): E16.5: control, 6.49×10^−4^±2.5×10^−5^ (n = 4 embryos); *Fgfr2* cKO, 4.43×10^−4^±2.4×10^−5^ (n = 4 embryos); E18.5: control, 9.41×10^−4^±5.9×10^−5^ (n = 3 embryos); *Fgfr2* cKO, 6.25×10^−4^±5.6×10^−5^ (n = 3 embryos); *Tnc* ^+^ cells in anterior EGL (N): E16.5: control, 3.00±0.41 (n = 4 embryos); *Fgfr2* cKO, 17.00±1.29 (n = 4 embryos); E18.5: control, 5.67±0.88 (n = 3 embryos); *Fgfr2* cKO, 13.33±2.33 (n = 3 embryos); Student's *t*-test). EGL, external granular layer; PCL, Purkinje cell layer; VZ, cerebellar ventricular zone. Scale bars: 100 µm (B); 50 µm (D,H); 30 µm (L).

The previous results indicated a strong correlation between the higher expression of *Fgfr2* in the developing anterior CbA, particularly in what appeared to be migrating glial cells (Figure 3), and the prominent anterior PC, GC and BG layering defects in the adult *Fgfr2* cKO cerebella (Figure 2). These defects are expected to arise between E14.5 and E16.5, because *Fgfr2* is not expressed in the CbA before E14.5 (our data and [19]). Indeed, initial defects were apparent at E16.5 in the CbA of the mutant embryos, and detected in 15 out of 28 (∼54%) *Fgfr2* cKO embryos. Double immunostaining for the neural progenitor marker Sox2, which is also expressed in RG/BG cells [46]–[48], and the RG/BG marker Blbp (also known as brain fatty acid binding protein 7, Fabp7) [7], [47], [49], [50], revealed a strong reduction of Sox2- and Blbp-expressing cells in the CbA of the *Fgfr2* cKO embryos at E16.5 and E18.5 (Figure 4A,D,G,I). A reduction of Sox2^+^ and Blbp^+^ neural progenitors and RG/BG precursors was also apparent in the anterior part of the cerebellar VZ of the mutant embryos at E16.5 (Figure 4A,D) whereas at E18.5, Sox2^+^ and Blbp^−^ neural progenitor cells appeared to accumulate in the cerebellar VZ of the mutant embryos (Figure 4G,I). Furthermore, only few Blbp^+^ RG/BG processes reached the pial surface of the mutant CbA, and these fibers were frequently arranged in a parallel (tangential) rather than perpendicular (radial) manner relative to this surface in the *Fgfr2* cKO embryos (Figure 4B,C,E,F,H,J). Notably, we also detected an increased number of ectopically positioned Sox2^+^/Blbp^+^ BG cells within the EGL of the mutant embryos compared with the control embryos at both stages (Figure 4B,C,E,F,H,J). Because the strong reduction of Sox2^+^ and Blbp^+^ cells already indicated a defective generation and/or differentiation of BG cells in the *Fgfr2* cKO embryos, we also determined the expression of Tenascin C (*Tnc*), an extracellular matrix glycoprotein whose mRNA is localized to the somata of RG precursors and BG cells and considered as one of the earliest marker for nascent BG [36], [47]. *Tnc* is transcribed in cells located in the cerebellar VZ, in single cells within the CbA, and in cells that begin to align within the PCL along the entire anterior-posterior extent of the CbA in E16.5–E18.5 control mice (Figure 5A,C,E,I). Only very few *Tnc*-expressing cells were detected within the EGL of control embryos at E16.5 and later stages (Figure 5A,C,E,I,N). In the CbA of the *Fgfr2* cKO embryos, by contrast, the numbers of *Tnc* ^+^ cells were strongly reduced already at E16.5 (Figure 5B,D,F,J,M). Moreover, many *Tnc*-expressing cells were ectopically positioned within the EGL of the mutant CbA (Figure 5B,D,F,J,N). Intensely Nissl-stained (glial) cells located within the forming PCL or migrating towards this layer showed an ISH signal for *Fgfr2* and *Tnc* in control embryos (Figure 5E,G,K,L). The ISH signal for *Fgfr2* was completely lost in the mutant CbA, and fewer intensely Nissl-stained and *Tnc*-expressing cells were detected within the forming PCL or en route towards this layer in the *Fgfr2* cKO embryos (Figure 5F,H). These results suggested that it is in fact the cell-autonomous loss of FGFR2 function in glial cells (RG and BG) that causes the defective generation of BG cells and their abnormal positioning within the EGL in the mutant embryos.

**Figure 6:**
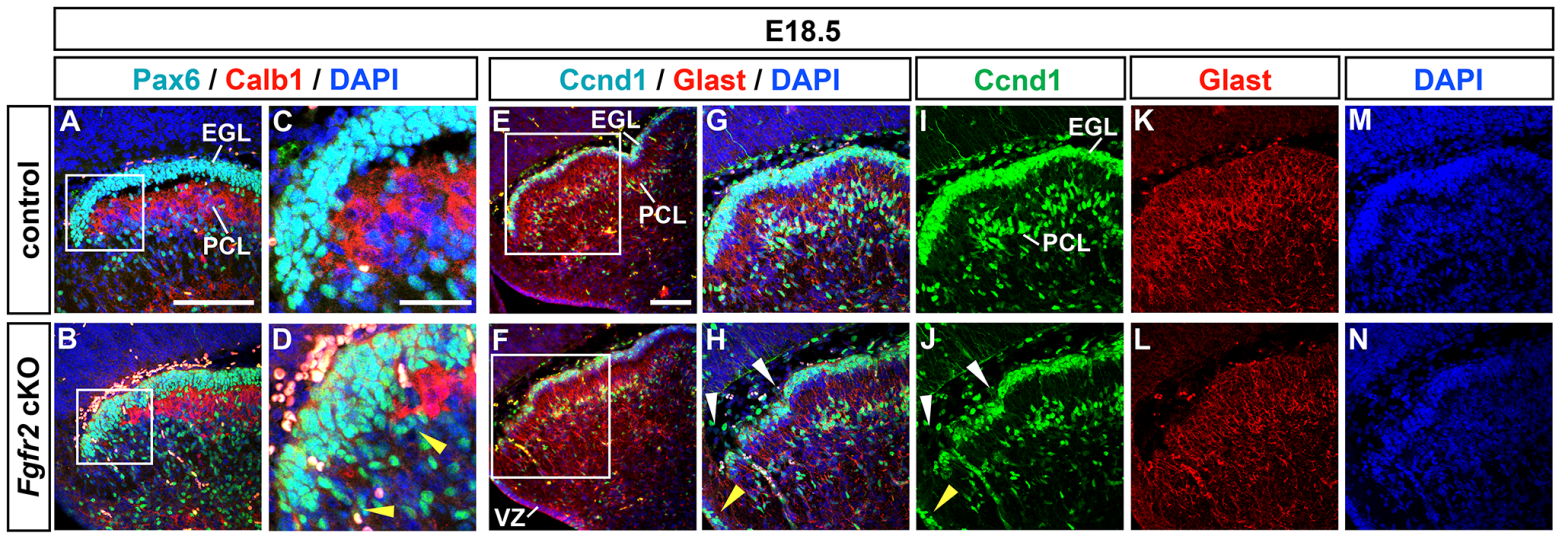
Disruption of the anterior PCL but apparently normal RG scaffold in the *Fgfr2* cKO CbA. (**A–N**) Representative confocal overviews (A,B,E,F) and close-up views (C,D,G–N) of the anterior CbA on sagittal sections from control (A,C,E,G,I,K,M) and *Fgfr2* cKO (B,D,F,H,J,L,N) embryos at E18.5 (n = 5 embryos/genotype), immunostained for Pax6 (cyan/green in A–D; a marker for GCPs) and Calb1 (red in A–D; a marker for PCs), or Ccnd1 (cyan/green in E–J; a marker for cycling GCPs and RG/BG precursors/cells) and Glast (red in E–H,K,L; a marker for RG/BG fibers), and counterstained with DAPI (blue in A–H,M,N; a nuclear marker). (C,D) and (G,H) are close-up views of the boxed areas in (A,B) and (E,F), respectively, (G,I,K,M) were taken from an adjacent section to the one shown in (E). (I–N) are single color channel views of (G,H), respectively. Yellow arrowheads in (D) delimit the lacking Calb1^+^ anterior PCL in the mutant embryos, and in (H,J) point at ectopically located Ccnd1^+^ RG/BG precursors within the mutant cerebellar VZ. White arrowheads in (H,J) delimit the distorted Ccnd1^+^ anterior outer EGL in the mutant embryos. EGL, external granular layer; PCL, Purkinje cell layer; VZ, cerebellar ventricular zone. Scale bars: 100 µm (A); 30 µm (C); 50 µm (E).

**Figure 7:**
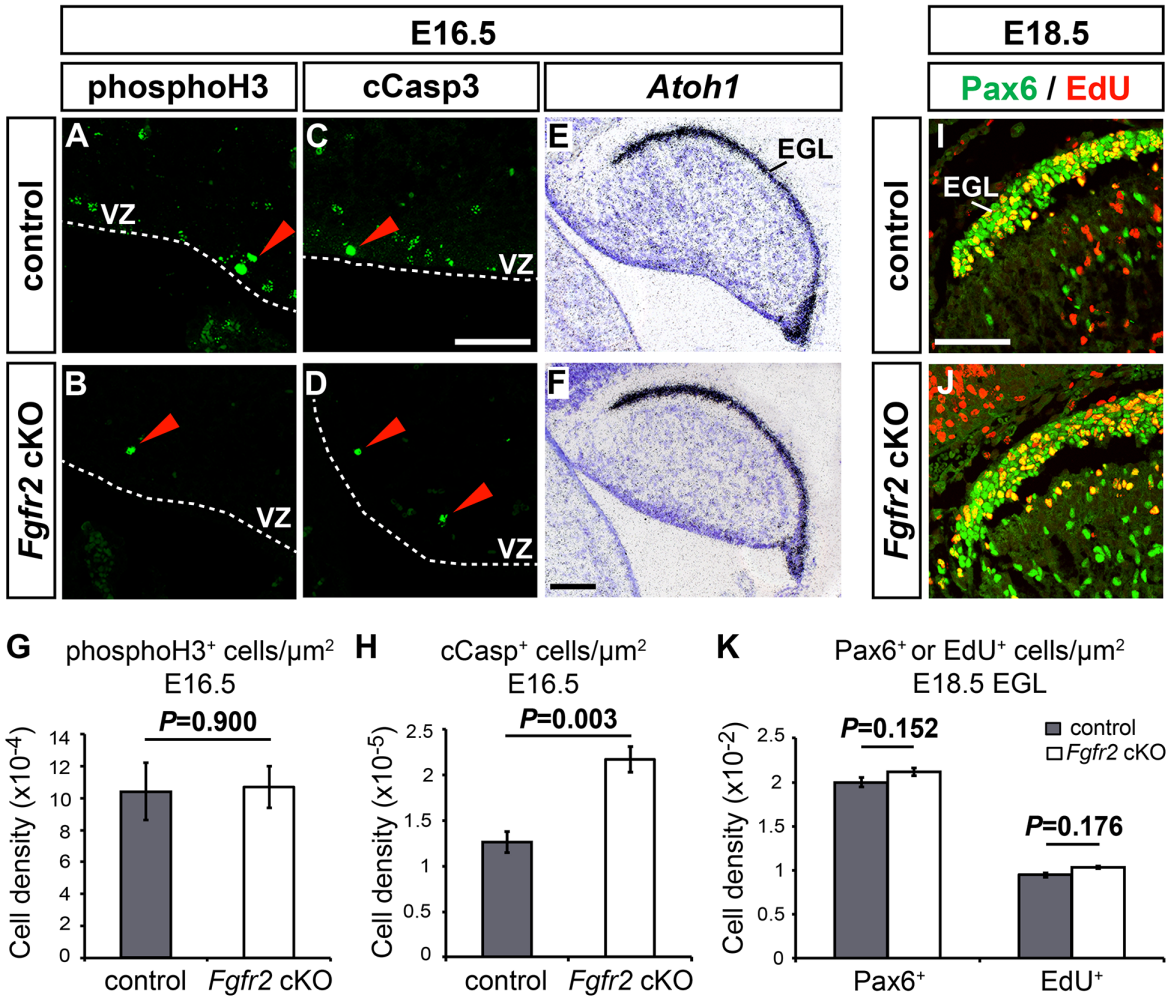
Reduced cell survival in the CbA of the *Fgfr2* cKO embryos. (**A–D**) Representative high magnification views of the VZ in the anterior CbA on sagittal sections of E16.5 control (A,C; n = 4 embryos) and *Fgfr2* cKO embryos (B,D; n = 4 embryos), immunostained for phosphorylated Histone H3 (phosphoH3) (A,B) and cleaved (activated) Caspase3 (cCasp3) (C,D). Red arrowheads point at phosphoH3^+^ or cCasp3^+^ cells, respectively. (**E,F**) Representative overviews of the CbA from E16.5 control (E; n = 4 embryos) and *Fgfr2* cKO embryos (F; n = 4 embryos), hybridized with a radioactive riboprobe for *Atoh1* (*Math1*). (**G,H**) Quantification of phosphoH3^+^ (G) and cCasp3^+^ (H) cell densities in the anterior CbA of E16.5 control (grey bars) and mutant (white bars) embryos (phosphoH3^+^ cells/µm^2^ (G): control, 10.4×10^−4^±0.18×10^−3^ (n = 4 embryos); *Fgfr2* cKO, 10.7×10^−4^±0.13×10^−3^ (n = 4 embryos); cCasp3^+^ cells/µm^2^ (H): control, 1.26×10^−5^±0.11×10^−5^ (n = 4 embryos); *Fgfr2* cKO, 2.17×10^−5^±0.15×10^−5^ (n = 4 embryos); Student's *t*-test). (**I,J**) Representative high magnification views of the EGL in the anterior CbA on sagittal sections of E18.5 control (I, n = 3 embryos) and *Fgfr2* cKO (J, n = 2 embryos) cerebella, immunostained for Pax6 (green) and EdU (red). (**K**) Quantification of Pax6^+^ GCP and proliferating EdU^+^ cell densities in the anterior EGL of E18.5 control (grey bars) and mutant (white bars) embryos (Pax6^+^ cells/µm^2^: control: 2.00×10^−2^±0.05×10^−2^ (n = 3 embryos); *Fgfr2* cKO: 2.11×10^−2^±0.02×10^−2^ (n = 2 embryos); EdU^+^ cells/µm^2^: control: 0.95×10^−2^±0.04×10^−2^ (n = 3 embryos); *Fgfr2* cKO: 1.03×10^−2^±0.01×10^−2^ (n = 2 embryos); Student's *t*-test). EGL, external granular layer; VZ, ventricular zone. Scale bars: 50 µm (C,I); 100 µm (F).

We next assessed whether the PCs (expressing Calb1, [51]) and GCPs (expressing Pax6 and Cyclin D1 (Ccnd1), [52]–[54]) had acquired their molecular identity and correct position within the developing CbA of the *Fgfr2* cKO embryos. At E18.5, Calb1^+^ PCs had not formed a multilayer underlying the most anterior (rostral) part of the Pax6^+^ EGL (Figure 6A–D), indicating a disrupted formation of the anterior PCL in the mutant embryos. Furthermore, Pax6^+^ GCPs were not aligned in a clearly delimited anterior EGL as in control embryos, and the mutant anterior EGL appeared to be slightly distorted with single Pax6^+^ GCPs protruding into the CbA (Figure 6A–D). In line with these observations, the arrangement of cycling Ccnd1^+^ GCPs within the outer EGL also appeared to be distorted in the anterior CbA of the *Fgfr2* cKO embryos (Figure 6E–J). Moreover, a reduced number of RG/BG precursors/cells expressing Ccnd1 [54], [55] was apparent in the mutant CbA at this stage, and some of these cells were ectopically located within the cerebellar VZ of the *Fgfr2* cKO embryos (Figure 6E–J). The glial high affinity glutamate transporter Glast (also known as Slc1a3 or Eaat1) is expressed in RG and BG cells and fibers [56]. Despite an apparently normal formation of the Glast^+^ RG fiber scaffold in the CbA of the mutant embryos, and concordantly with the decreased Ccnd1^+^ cells in this region, the Glast^+^ signal appeared to be reduced in the mutant PCL (Figure 6K,L). Because the PC and GCP defects appeared only at around E17.5 (Figure S4 in File S1; see also Figure 7 for *Math1* (*Atoh1*) detection in the E16.5 EGL), i.e. at least one day after the BG defects were detected in the *Fgfr2* cKO embryos, we concluded that the reduced numbers of RG/BG precursors/cells and the ectopic positioning of BG cells in the mutant EGL are the primary cerebellar defects in the *Fgfr2* cKO embryos.

### Reduced cell survival in the anterior CbA of the *Fgfr2* cKO embryos

The reduced numbers of Sox2^+^/Blbp^+^ and *Tnc* ^+^ RG/BG precursors/cells, and the disrupted formation of the presumptive anterior PCL, suggested that the proliferation of the BG and/or PC progenitors located in the VZ of the mutant CbA and/or their survival might also be affected in the absence of *Fgfr2*. The latter possibility was more likely in the *Fgfr2* cKO embryos because *Fgfr2* is not expressed in the cerebellar VZ and *Fgfr1* expression was not altered in this region of the mutant CbA (Figure 3). Indeed, the numbers of mitotic (pH3^+^) cells in the VZ of the CbA were not significantly different between control and *Fgfr2* cKO embryos at E16.5 (Figure 7A,B,G), suggesting that the proliferation of BG and/or PC progenitors was not affected in the mutant embryos at this stage. The numbers of apoptotic (cCasp3^+^) cells, by contrast, were significantly increased by ∼1.7-fold in the anterior CbA of the *Fgfr2* cKO embryos at E16.5 (Figure 7C,D,H), indicating that cell survival within the CbA was compromised in the mutant embryos. The increased apoptotic cell death in the anterior CbA of the *Fgfr2* cKO embryos coincided with a slight but not significant decrease of the total area of the mutant CbA by ∼9% at E16.5 (control: 10.02×10^5^±0.64×10^5^ µm^2^; *Fgfr2* cKO: 9.14×10^5^±0.73×10^5^ µm^2^; *P* = 0.40 Student's *t*-test) and ∼10% at E18.5 (control: 3.01×10^6^±0.26×10^6^ µm^2^; *Fgfr2* cKO: 2.73×10^6^±0.15×10^6^ µm^2^; *P* = 0.40 Student's *t*-test). Because the increased (∼1.7-fold) number of apoptotic cells corresponded with the decreased (∼1.5-fold) number of *Tnc* ^+^ cells, we concluded that the reduced number of RG/BG precursors/cells in the mutant CbA might also be due to a reduced survival of these cells in the absence of FGFR2 signaling.

The generation of the EGL and GCPs was not affected in the *Fgfr2* cKO embryos, as determined by the normal expression of *Atoh1* (*Math1*), a transcription factor required for EGL and GCP development [57], in the developing mutant CbA at E16.5 and E18.5 (Figure 7E,F and data not shown). We also determined whether the proliferation and cell-cycle exit of GCPs in the anterior (rostral) EGL might have been affected in the *Fgfr2* cKO embryos. The density of Pax6^+^ GCPs and proliferating (EdU^+^) cells in the anterior EGL (Figure 7I–K), as well as the fraction of proliferating Pax6^+^ GCPs that had incorporated EdU after a single pulse given 24 h before (EdU^+^ and Pax6^+^ cells per total Pax6^+^ cells: control, 47.5±2.7%; *Fgfr2* cKO, 48.9±0.1%; *P* = 0.65 in the Student's *t*-test), were not significantly different between control and mutant embryos at E18.5, indicating that the numbers and the proliferation/cell-cycle exit of Pax6^+^ GCPs in the anterior EGL were not affected by the loss of *Fgfr2* expression in the CbA. Together, the previous results suggested that FGFR2-mediated signaling is also required for the proper survival of RG/BG precursors/cells and PCs within the CbA.

### FGF target gene activation is almost completely abolished in the CbA of the *Fgfr2* cKO embryos

**Figure 8:**
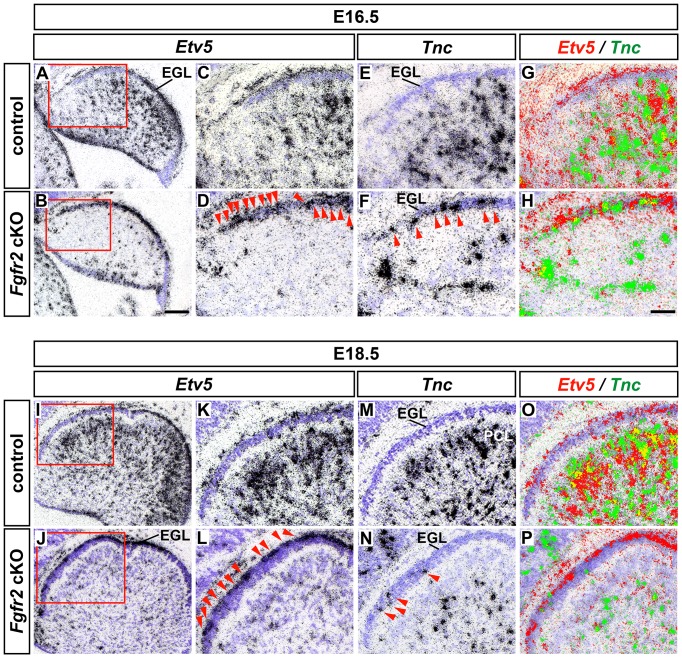
FGF target gene activation is almost completely abolished in the CbA of *Fgfr2* cKO embryos. (**A–P**) Representative sagittal brightfield views of E16.5 (A–H; n = 5 embryos/genotype) and E18.5 (I-P; n = 4 embryos/genotype) control (A,C,E,G,I,K,M,O) and *Fgfr2* cKO (B,D,F,H,J,L,N,P) cerebella, hybridized with riboprobes for *Etv5* (A–D,I–L) and *Tnc* (E,F,M,N). (C,D) and (K,L) are higher magnifications of the boxed areas in (A,B) and (I,J), respectively. (G,H) and (O,P) are pseudo-colored overlays (*Etv5* in red, *Tnc* in green, overlapping expression domains appear in yellow) of the adjacent sections shown in (C–F) and (K–N), respectively. Red arrowheads in (D,F,L,N) point at ectopic *Etv5* ^+^ (D,L) or *Tnc* ^+^ (F,N) cells in the anterior EGL of the mutant embryos. Note that at E18.5, the ectopic *Etv5* ^+^ cells are predominantly located in the outer margin of the EGL, whereas the ectopic *Tnc* ^+^ cells are mostly confined to the inner EGL. EGL, external granular layer; PCL, Purkinje cell layer. Scale bars: 100 µm (B); 50 µm (H).

To confirm that FGF signaling was in fact reduced or abolished in those regions of the mutant CbA where *Fgfr2* is highly expressed (anterior CbA including the anterior PCL but excluding the VZ, see Figure 3), we determined the transcription of a known FGF target gene, *Etv5* (*Erm*, [19], [58]) in the developing CbA of control and *Fgfr2* cKO embryos. At E16.5 and E18.5, *Etv5* is strongly expressed in the cerebellar VZ, in scattered cells within the CbA, in the emerging PCL and in the posterior EGL of control embryos (Figure 8A,I; [19]). By contrast, transcription of *Etv5* was strongly reduced in almost the entire CbA (including the VZ and the PCL primordium but excluding the posterior EGL) of the mutant embryos at E16.5, and was almost completely abolished at E18.5 (Figure 8B,J). Notably, *Etv5* was ectopically expressed in the anterior EGL of the *Fgfr2* cKO embryos at both stages (Figure 8C,D,K,L). The ectopic *Etv5*-expressing cells clearly outnumbered the ectopically positioned *Tnc* ^+^ cells in the anterior EGL of the mutant embryos (Figure 8C–F,K–N). Moreover, the ectopic *Etv5* ^+^ cells were largely confined to the outer EGL at E18.5 (Figure 8L,P), whereas the ectopic *Tnc* ^+^ cells were predominantly located in the inner EGL at this stage (Figure 8N,P), indicating that FGF signaling was ectopically activated in cells other than the ectopically positioned *Tnc* ^+^ BG cells in this region of the mutant CbA. These results showed that FGF signaling and target gene activation were in fact strongly reduced or even abolished within the CbA, in the cerebellar VZ and in the forming PCL of the *Fgfr2* cKO embryos, thus including regions where *Fgfr2* is not or only weakly expressed (the VZ and posterior CbA/PCL, see Figure 3). As this correlated with an apparent reduction of *Fgfr1* expression in the mutant CbA (including the emerging PCL, Figure 3), we concluded that the lack of FGFR2-mediated signaling makes a major contribution to the BG and PC defects in the *Fgfr2* cKO embryos, but might additionally be reinforced by a reduction of FGFR1-mediated signaling in these embryos.

We next determined whether the relatively subtle PCL and EGL defects in the *Fgfr2* cKO embryos might be due to an altered expression of *Shh* in PCs or a defective SHH signal transduction in GCPs. The transcription of *Shh* and the SHH target gene *Ptch1* was not changed in the developing CbA of the mutant embryos compared with control embryos, except for a notable lack of the *Shh* ^+^ PCL underlying the most anterior (rostral) EGL in the *Fgfr2* cKO embryos at E18.5 (Figure S5 in File S1). The latter observation is most likely due to the lack of Calb1^+^ PCs in the most anterior PCL of the mutant embryos (see Figure 6). Together, these results suggested that the expression of *Shh* in migrating and stationary PCs and its target gene *Ptch1* in GCPs is not altered by the reduced or even abolished FGF signaling in the mutant CbA, and is therefore unlikely to contribute to the PCL and EGL defects in the *Fgfr2* cKO embryos.

### FGF9/FGFR signaling inhibits the migration of RG/BG precursors/cells in cerebellar microexplants *in vitro*

**Figure 9:**
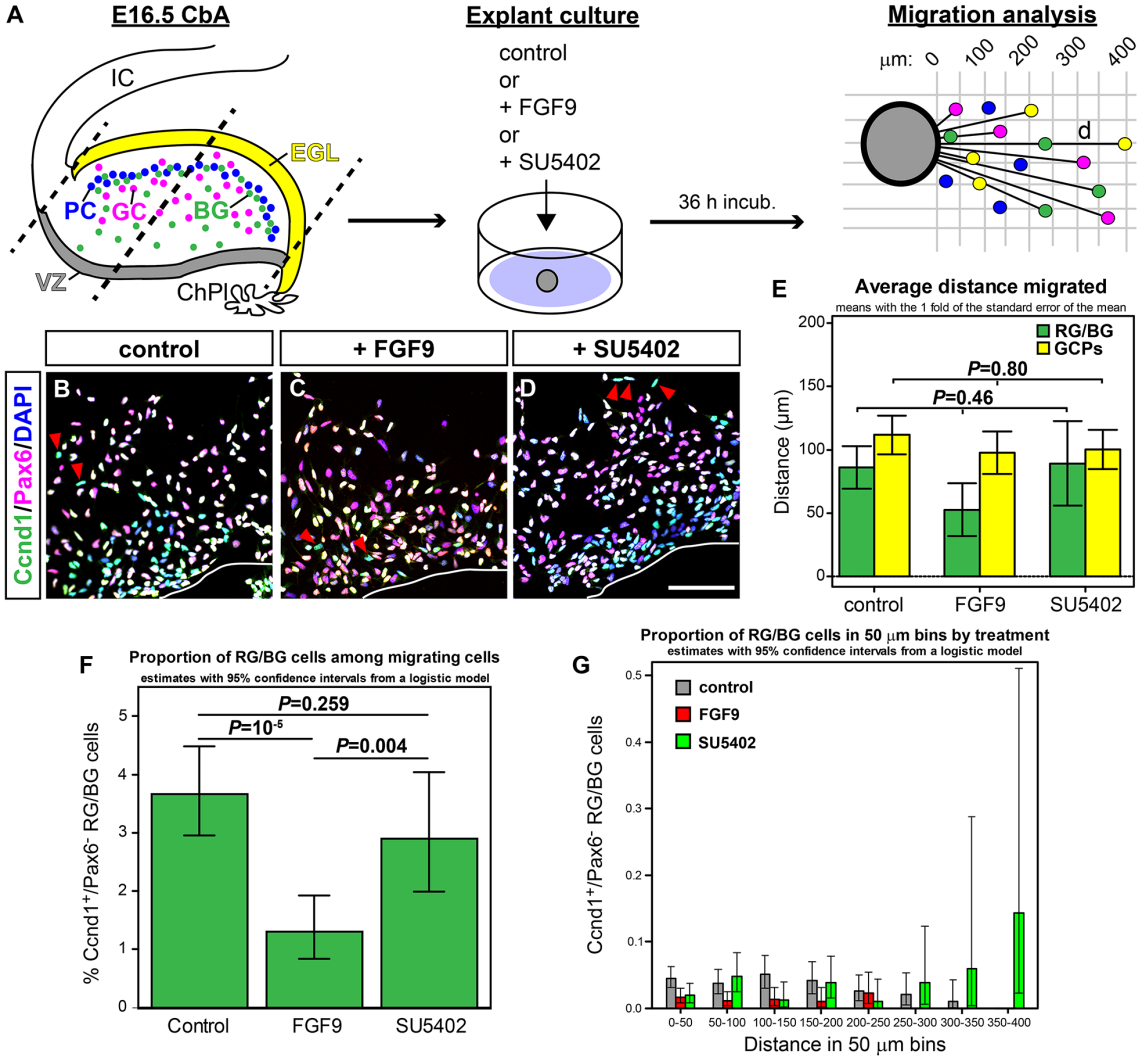
FGF9/FGFR signaling inhibits the migration of RG/BG precursors/cells in cerebellar microexplants *in vitro*. (**A**) Migration assays were performed with similarly sized (black dotted bars) CbA microexplants from E16.5 wild-type (CD-1) mice, containing Pax6^+^/Ccnd1^+^ GCPs in the outer EGL (yellow), Ccnd1^+^ RG/BG precursors and cells (green), Pax6^+^ postmitotic GCs and inner CbA cells (pink), and DAPI^+^ PCs (blue). ChPl, choroid plexus; EGL, external granular layer; IC, inferior colliculus; VZ, cerebellar ventricular zone. CbA microexplants were treated with control medium or medium containing 100 ng/ml FGF9 or 20 µM SU5402. The distance (d) migrated by each Pax6^−^/Ccnd1^+^ (green), Pax6^+^/Ccnd1^+^ (yellow) and Pax6^+^/Ccnd1^−^ (pink) cell from the border of the microexplant was measured after 36 h of incubation. (**B–D**) Representative confocal overviews of Ccnd1^+^ (green) and/or Pax6^+^ (red) cells (double-positive cells appear in yellow), counterstained with DAPI (blue) (overlays with single-positive cells appear in light green and pink, respectively), that migrated from the border of the CbA microexplant (white line) in control (B), FGF9- (C) or SU5402- (D) containing medium. Red arrowheads point at the front-most Ccnd1^+^/Pax6^−^ (green) cells. (**E**) Quantification of the average distance migrated by RG/BG precursors/cells (green bars) and GCPs (yellow bars) in control- (n = 8 explants), FGF9- (n = 8 explants) or SU5402- (n = 6 explants) treated microexplant cultures (Distance (µm): RG/BG precursors/cells, control, 86.2±16.8; +FGF9, 52.6±20.9; +SU5402, 89.3±33.2; GCPs, control, 111.7±15.3; +FGF9, 97.7±16.8; +SU5402, 100.2±15.6; one-way ANOVA). (**F**) Quantification of the proportion of Ccnd1^+^/Pax6^−^ RG/BG precursors/cells among the total number of migrating Ccnd1^+^ and/or Pax6^+^ cells in control-, FGF9- or SU5402-treated microexplant cultures (% Ccnd1^+^/Pax6^−^ RG/BG precursors/cells: control, 3.66, 95% confidence interval [2.95,4.48] (8 experiments for controls with 128–589 migrated cells, among them 2-29 RG/BG cells); +FGF9, 1.31, 95% confidence interval [0.84,1.92] (8 experiments for FGF9 with 79-501 migrated cells, among them 0–9 RG/BG cells); +SU5402, 2.89, 95% confidence interval [1.99,4.04] (6 experiments for SU5402 with 78–371 migrated cells, among them 0–15 RG/BG cells); *P*-values from contrasts of a logistic model). (**G**) Average proportions of Ccnd1^+^/Pax6^−^ RG/BG precursors/cells among the total number of migrating Ccnd1^+^ and/or Pax6^+^ cells in each 50-µm bin in control- (grey bars), FGF9- (red bars) or SU5402- (green bars) treated microexplant cultures were estimated with a logistic model. Values are given in Table S2 in File S1. Scale bar (D): 100 µm.

In addition to the loss of RG/BG precursors/cells, the radial migration of Sox2^+^/Blbp^+^ and *Tnc* ^+^ BG cells within the CbA did not stop at the level of the PCL, leading to the ectopic positioning of BG cells within the prenatal EGL or adult ML, respectively, of the *Fgfr2* cKO mice (Figures 2, 4, 5). This suggested that FGFs secreted from the EGL and/or PCL inhibit the further migration of RG/BG precursors/cells, thereby controlling their correct alignment within the developing PCL. To test this hypothesis, we prepared CbA microexplant cultures from E16.5 wild-type (CD-1) embryos and treated them for 36 h with control medium or medium containing recombinant human FGF9 or SU5402 (Figure 9A). FGF9 is an FGF expressed by GCPs and PCs, and required for the proper differentiation and alignment of BG cells in the mouse cerebellum [19], [20], whereas SU5402 is a known inhibitor of FGFR signaling [59]. After immunocytochemical staining for Pax6, a marker for GCPs and migrating GCs as well as other cells located within the CbA (Figures 6A, 9A, S4 in File S1; [52]), and Ccnd1 (Cyclin D1), which is expressed in proliferating GCPs from the outer EGL and in RG/BG precursors/cells within the CbA and forming PCL but not in VZ progenitors (Figures 6E, 9A, S4 in File S1; [53]–[55]), we determined the distance migrated by each cycling Pax6^+^/Ccnd1^+^ GCP and Pax6^−^/Ccnd1^+^ RG/BG precursor/cell or postmitotic Pax6^+^/Ccnd1^−^ GC from the border of the microexplant (Figure 9A). The average distance migrated by Pax6^+^/Ccnd1^+^ GCPs did not show any notable differences between control-, FGF9- and SU5402-treated cultures (Figure 9B–E), whereas the average distance migrated by Pax6^−^/Ccnd1^+^ RG/BG precursors/cells was reduced after FGF9 treatment and slightly increased after SU5402 treatment relative to the control-treated cultures, although it did not reach statistical significance because of the high variance of migrated distances within and between experiments (Figure 9B–E). Because this already hinted at a migration-inhibiting effect of FGF9 and migration-promoting effect of the FGFR inhibitor SU5402 on RG/BG precursors/cells but not on GCPs, we analyzed the migratory behavior of RG/BG precursors/cells under these conditions in more detail. FGF9 treatment reduced significantly the proportion of Pax6^−^/Ccnd1^+^ RG/BG precursors/cells among the total number of Ccnd1 and Pax6 single- and double-positive cells (including Pax6^−^/Ccnd1^+^ RG/BG, Pax6^+^/Ccnd1^+^ GCPs and Pax6^+^/Ccnd1^−^ GCs, Figures 6A, 6E, 9A) that had migrated from the core of the microexplant (regardless of distance) relative to the control- and SU5402-treated explants (Figure 9F), indicating that FGF9 in fact inhibited the outward migration of RG/BG precursors/cells in these cultures. Next, we assessed the proportion of Pax6^−^/Ccnd1^+^ RG/BG precursors/cells among all Ccnd1 and Pax6 single- and double-positive cells in 50-µm bins from the border of the microexplants (Figure 9A,G; Table S2 in File S1). We noted that under control conditions, the average proportion of RG/BG precursors/cells in each bin corresponded well with a normal distribution according to the distance migrated, with fewer or no cells in the more proximal and distal bins, respectively, relative to the border of the microexplants (Figure 9B,G). After FGF9 treatment, however, the average proportion of RG/BG precursors/cells in each bin was strongly decreased and these cells were only detected up to a distance of 200–250 µm from the border of the microexplants (Figure 9C,G), indicating that the distance migrated by the RG/BG precursors/cells was also reduced in the FGF9-treated cultures. Treatment of the microexplants with SU5402, by contrast, strongly increased the average proportion of RG/BG precursors/cells particularly in the more distal bins and these cells were detected up to a distance of 350–400 µm from the border of the microexplants (Figure 9D,G), indicating that the inhibition of FGFR signaling augmented the distance migrated by the RG/BG precursors/cells *in vitro* in a similar manner to what was observed in the *Fgfr2* cKO embryos *in vivo* (Figures 4,5). Together, these data showed that FGF9 inhibits the outward migration of RG/BG precursors/cells whereas blocking FGFR signal transduction has the opposite effect and promotes the outward migration of these cells for longer distances from the microexplant, and thus strongly suggested that during cerebellar development, FGF9/FGFR2-mediated signaling in migrating RG/BG precursors/cells inhibits their further migration beyond their proper position within the PCL.

## Discussion

We show here that the conditional inactivation of *Fgfr2* in neural progenitors results in specific cellular and layering defects in particular in the anterior (rostral) cerebellum of adult *Fgfr2* cKO mice, where *Fgfr2* is highly expressed during normal CbA development. The developmental deficits in these mice include a reduced generation and ectopic positioning of BG cells within the EGL, the misalignment and lack of PCs in the most anterior PCL, and a reduced cell survival in the developing CbA. We also show that FGF9/FGFR2-mediated signaling inhibits the outward migration of RG/BG precursors/cells *in vitro*, and might thereby control the correct positioning of BG cells within the PCL *in vivo*.

### FGFR2-mediated signaling promotes the generation of BG cells and cell survival in the developing CbA

Adult *Fgfr2* cKO mice displayed similar but generally weaker cerebellar defects than previously described in *Fgfr1*/*Fgfr2* double mutant (*Nestin-Cre*;*Fgfr1^flox^*;*Fgfr2^flox^* [20] and *hGFAP-Cre*;*Fgfr1^f/f^*;*Fgfr2^f/f^* [21]) mice. Therefore, we determined the relative contribution of the lack of *Fgfr2* to these phenotypes. Our results indicated that *Fgfr2* transcription in the developing CbA starts after E14.5, is highest in cells located in its anterior (rostral) part including the anterior PCL, and spares the cerebellar VZ. *Fgfr1*, by contrast, is transcribed at high levels in a much broader area including the posterior CbA/PCL and the entire cerebellar VZ [19]. The anterior CbA gives rise to the anterobasal lobe around birth, which generates the anterior lobules (lobuli I–III) of the adult cerebellum [1]; correspondingly, these lobules were the most affected in the adult *Fgfr2* cKO cerebellum. A stronger phenotype in the anterior cerebellum was also noted in FGF signaling loss-of-function (LOF) (*hGFAP-Cre*;*Fgfr1^f/f^*;*Fgfr2^f/f^* [21]) and gain-of-function (GOF) (*En1^cre/+^*;*Spry1^flox/flox^*;*Spry2^flox/flox^* [22]) mutant mice, suggesting that the restricted high expression of *Fgfr2* in the anterior CbA imposes a stronger need of a balanced FGF signaling for the proper development of this region. Nevertheless, the strongly reduced or even abolished transcription of the FGF target gene *Etv5* in the entire CbA (except the EGL) of the *Fgfr2* cKO embryos, including the cerebellar VZ and posterior CbA regions where *Fgfr2* is not expressed at detectable high levels, suggests a contribution of the apparently decreased *Fgfr1* expression to the cerebellar phenotypes of the *Fgfr2* single mutant mice (see comments below).

The most notable defect in the developing CbA of the *Fgfr2* cKO embryos was a strong reduction of Sox2-, Blbp- and *Tnc*-expressing BG precursors and cells that became apparent already at E16.5, i.e. less than two days after the failed induction of *Fgfr2* expression in the corresponding neural progenitors. Sox2^+^ neural progenitors indeed appeared to accumulate over time in the cerebellar VZ of the *Fgfr2* cKO embryos, suggesting that these cells failed to generate the proper amount of migrating Blbp^+^ and *Tnc* ^+^ BG precursors/cells in the absence of *Fgfr2*. A similar albeit much stronger BG phenotype was recently described in conditional mouse mutants for the protein tyrosine phosphatase, non-receptor type 11 gene (*Ptpn11*, also known as *Shp2*), an intracellular effector of the FGF/FGFR signaling pathway (*En1*;*Ptpn11^CKO^* mice, [47]). The generation of BG is completely abolished in these mice, apparently because RG fails to transform into BG in the absence of *Ptpn11*, which subsequently leads to foliation defects in the mutant cerebella [47]. Our results thus suggest that FGFR2 is primarily involved in the transduction of FGF signals required for the proper transformation and/or differentiation of RG precursors into BG cells.

We also observed a slightly increased number of apoptotic cells within the CbA of the *Fgfr2* cKO embryos. The reduced cell survival most likely includes migrating and stationary PC and RG/BG precursors/cells that are born at earlier developmental stages in the cerebellar VZ, and might contribute to the reduced numbers of Sox2^+^/Blbp^+^/*Tnc* ^+^/S100b^+^ RG/BG precursors/cells and to the lack of Calb1^+^ PCs in the anterior PCL of the embryonic and adult *Fgfr2* cKO cerebellum. BG cells are also decreased in the *hGFAP-Cre*;*Fgfr1^f/f^*;*Fgfr2^f/f^* cerebellum, although apoptotic cell numbers do not appear to be changed in these mice [21]. In contrast to cell survival, the proliferation of cerebellar VZ progenitors was not affected in the *Fgfr2* cKO embryos. Although we cannot exclude that FGFR2 might control the proliferation of migrating RG/BG precursors, including those generating the prospective BG cells [13], FGFR2-mediated signaling is unlikely to control the prenatal proliferation of VZ progenitors for several reasons: 1) *Fgfr2* is not transcribed at detectable levels in the cerebellar VZ throughout embryonic development; 2) PCs are born at E10-13 in the mouse [1], [60], long before *Fgfr2* expression initiates in the CbA (after E14.5); 3) The onset of *Fgfr2* transcription in the CbA coincides with the peak of BG radial migration toward the PCL at E15 in the mouse [13].

In contrast to the *hGFAP-Cre*;*Fgfr1^f/f^*;*Fgfr2^f/f^* [21] and *Nestin-Cre*;*Fgf9^flox^* [20] mice, we did not detect any defects in GCP numbers and proliferation in our *Fgfr2* cKO mice. This is consistent with the lack of *Fgfr2* transcription in the EGL of the wild-type embryo, and coincides with an ectopic activation of FGF signaling (assessed by *Etv5* expression) in the anterior EGL of the *Fgfr2* cKO embryos. Notably, the ectopic *Etv5*-expressing cells did not overlap with the ectopically positioned *Tnc* ^+^ BG cells in this region of the mutant CbA. This finding suggests either that the ectopic *Etv5*-expressing cells derived from the *Etv5* ^+^ posterior EGL and failed to downregulate the expression of *Etv5* during their tangential migration towards the anterior (rostral) EGL, or that FGF signaling was ectopically activated in these cells by an unknown, non-cell-autonomous mechanism in the absence of *Fgfr2*. Furthermore, PCs were ectopically positioned within the GL and GCs “protruded” into the PCL in the anterior lobules of the adult *Fgfr2* cKO cerebellum. The apparently normal alignment of Glast^+^ RG fibers in the CbA of the mutant embryos suggests that these are most likely secondary phenotypes appearing during postnatal cerebellar development in the *Fgfr2* cKO mice. The disruption of the Blbp^+^ and Gfap^+^ BG fiber scaffold in the mutant cerebellum might thus lead to an aberrant alignment of single PCs within the PCL, and to the blocked migration of GCs along these fibers through the PCL into the GL.

### Incomplete penetrance and *Fgfr1* as a genetic modifier of the *Fgfr2* cKO cerebellar phenotype

The cerebellar defects of the *Fgfr2* cKO mice are not completely penetrant and may have been missed inadvertently in previous analyses of *Fgfr2* single mutant mice [20], [21]. Because cerebellar phenotypes are particularly sensitive to genetic backgrounds [61], it is very likely that the incomplete penetrance of the *Fgfr2* cKO cerebellar phenotype is due to genetic modifiers in the mixed genetic background of our mice [62]. Indeed, the transcription of *Fgfr1* also appeared to be partially decreased in the CbA of the affected *Fgfr2* cKO embryos. As this was also true for regions within the CbA where *Fgfr2* is not expressed at high levels or in many cells (such as the posterior CbA/PCL), the reduced expression of *Fgfr1* might be one genetic modifier in these mice. Alternatively, the loss of FGFR2 function might affect the transcription of *Fgfr1* cell-autonomously or non-cell-autonomously by yet unknown mechanism(s) in the developing CbA. Another reason for the different cerebellar phenotypic outcomes of our *Fgfr2* cKO and the previously generated conditional *Fgfr2* mutant mice might be the different gene targeting strategies used for generating these mice [27], [63], although they should all result in the absence of a functional FGFR2 receptor in the developing CbA.

FGF9/FGFR2-mediated signaling might act as a positioning cue for migrating BG cells

BG cells were located ectopically in the anterior EGL/ML of the *Fgfr2* cKO cerebella, indicating that FGFR2 signaling is necessary for their proper positioning within the PCL. The radial migration of BG precursors and cells from the VZ toward the PCL starts at ∼E14 and reaches a peak between E15–16 in the mouse [13], [36], the time interval when *Fgfr2* transcription initiates in the developing CbA. SHH secreted from PCs is a potent chemo-attractant for BG cells that strongly promotes their migration [15]. The normal transcription of *Shh* in PCs of the mutant CbA suggests that this guidance cue is not affected in the *Fgfr2* cKO embryos. The migration of BG cells, however, must be inhibited once these cells have reached their final destination in the PCL to prevent their ectopic positioning beyond this layer [13]. We therefore hypothesized that an FGF signal emitted from the EGL and/or PCL might provide such a “stop signal” to migrating BG cells. One potential candidate was FGF9 expressed in GCPs and PCs and required for the proper positioning of BG cells in the PCL [19], [20], although other FGFs expressed within the EGL (FGF1/10) or PCL (FGF4/15) [19] might have a similar function. The outward migration of RG/BG precursors/cells from CbA microexplants was indeed inhibited after FGF9 treatment of these explants, whereas FGFR blockade promoted the outward migration of RG/BG precursors/cells for longer distances from the explants. These results strongly suggest that RG/BG precursors/cells fail to detect the probably concentration-dependent FGF9 “stop signal” from the EGL/PCs in the absence of FGFR2-mediated signaling, and therefore migrate beyond their normal position within the PCL. Altogether, our findings thus reveal the specific pro-differentiation, anti-apoptotic and cell positioning functions of FGFR2-mediated signaling in RG/BG precursors/cells during cerebellar development in the mouse, and might provide new mechanistic insights to the pathogenesis of cerebellar ataxias.

## Supporting Information

File S1 **Table S1. Locomotor behaviors of control and** ***Fgfr2*** **cKO mice.** 12 weeks old male *Fgfr2^lox/lox^* (control, n = 15) and *Nestin-Cre;Fgfr2^lox/lox^* (*Fgfr2* cKO, n = 12) mice were tested in the modified hole board (mHB) for horizontal and vertical locomotor abilities. Motor coordination and balance was assessed with the rotating rod apparatus (Rotarod). All values given are mean ± s.e.m. **Table S2. Average proportion of Ccnd1^+^/Pax6^−^ RG/BG precursors/cells among the total number of migrating Ccnd1^+^ and/or Pax6^+^ cells in each 50-µm bin in control-, FGF9- or SU5402-treated microexplant cultures.** Values represent the average proportion of Ccnd1^+^/Pax6^−^ RG/BG precursors/cells among the total number of migrating Ccnd1^+^ and/or Pax6^+^ cells in each 50-µm bin (distance migrated from the border of the microexplant) and for each treatment, and the 95% confidence interval estimated with a logistic model (in 8 bins and 3 treatments: total cells migrated: 1168, among them RG/BG precursors/cells: 146). **Figure S1. Correlation of locomotor and cerebellar phenotypes in adult** ***Fgfr2*** **cKO mice.** (**A**) Rotarod performance (latencies to fall) of 15 control and 12 *Fgfr2* cKO males. Highlighted in red are the control male with the shortest latency to fall (16 sec, ID 30064154), and two *Fgfr2* cKO males with the longest (140 sec, ID 30064156) and shortest (25 sec, ID 30064164) latencies to fall. (**B–G**) Sagittal cerebellar sections from the adult males highlighted in red in (A), counterstained with DAPI (B,D,F) or immunostained for Calb1 (C,E,G). Note the severe cerebellar defects in the ID 30064164 *Fgfr2* cKO male with the shortest latency to fall from the Rotarod (F,G). I-X, lobuli of the adult cerebellum. Scale bar (B): 500 µm. **Figure S2. The ventral mid-/hindbrain region is not affected in** ***Fgfr2*** **cKO mice.** (**A–F**) Sagittal views of the ventral MHR from adult control (A,C,E) and *Fgfr2* cKO mice (B,D,F), hybridized with riboprobes for Tyrosine hydroxylase (*Th*; the rate-limiting enzyme for dopamine and noradrenaline synthesis) (A,B), the Serotonin transporter (*Sert*, also known as *Slc6a4*, expressed on serotonergic neurons) (C,D) and the vesicular Acetylcholine transporter (*VAChT*, also known as *Slc18a3*, expressed in cholinergic neurons) (E,F). Gross anatomical alterations of these neuronal populations were not detected in the *Fgfr2* cKO mice. DR, dorsal raphe nucleus; LC, locus ceruleus; LDTg, laterodorsal tegmental nucleus; RF, reticular formation (brainstem); SNc, substantia nigra pars compacta; VTA, ventral tegmental area. Scale bar (A): 500 µm. **Figure S3.** ***Fgfr*** **expression in the developing murine mid-/hindbrain region.** (**A–R**) Representative brightfield (A–C,G–I,M–O) and darkfield (D–F,J–L,P–R) views of the mid-/hindbrain region on cresyl-violet-stained midsagittal sections from wild-type (CD-1) mouse embryos at E14.5 (A–F; n = 5 embryos), E16.5 (G–L; n = 5 embryos), and E18.5 (M–R; n = 6 embryos), hybridized with radioactive *Fgfr1* (A,D,G,J,M,P), *Fgfr2* (B,E,H,K,N,Q) and *Fgfr3* (C,F,I,L,O,R) riboprobes. CbA, cerebellar anlage; ChPl, choroid plexus; EGL, external granular layer; IC, inferior colliculus; PCL, Purkinje cell layer; rH, rostral hindbrain; rl, rhombic lip; Tg, tegmentum; VZ, cerebellar ventricular zone. Scale bar (C): 200 µm. **Figure S4. Disruption of the anterior PCL but apparently normal RG scaffold in the E17.5** ***Fgfr2*** **cKO CbA.** (**A–L**) Representative confocal overviews (A,B,E–L) and close-up views (C,D) of the anterior CbA on sagittal sections from control (A,C,E,G,I,K) and *Fgfr2* cKO (B,D,F,H,J,L) embryos at E17.5 (n = 1 embryo/genotype), immunostained for Pax6 (cyan/green in A–D; a marker for GCPs) and Calb1 (red in A–D; a marker for PCs), or Ccnd1 (cyan/green in E–H; a marker for cycling GCPs and RG/BG precursors/cells) and Glast (red in E,F,I,J; a marker for RG/BG fibers), and counterstained with DAPI (blue in A–F,K,L; a nuclear marker). (C,D) are close-up views of the boxed areas in (A,B). (G–L) are single color channel views of (E,F), respectively. Yellow arrowheads in (D) delimit the lacking Calb1^+^ anterior PCL in the mutant embryos, and in (F) point at ectopically located Ccnd1^+^ RG/BG precursors within the mutant cerebellar VZ. White arrowheads in (F,H) delimit the distorted Ccnd1^+^ anterior outer EGL in the mutant embryos. EGL, external granular layer; PCL, Purkinje cell layer. Scale bars: 100 µm (A); 30 µm (C). **Figure S5. SHH signaling does not appear to be affected in the CbA of** ***Fgfr2*** **cKO embryos**. (**A–H**) Representative sagittal darkfield (A,B) and brightfield (C–H) views of the CbA in E16.5 (A–D; n = 5 embryos/genotype) and E18.5 (E–H; n = 4 embryos/genotype) control (A,C,E,G) and *Fgfr2* cKO (B,D,F,H) embryos, hybridized with riboprobes for *Shh* (A,B,E,F) and *Ptch1* (C,D,G,H). Red arrowheads in (F) delimit the lacking *Shh* ^+^ anterior PCL in the mutant embryos. EGL, external granular layer; PCL, Purkinje cell layer. Scale bar (A): 100 µm.
(PDF)
