# Supplementary material for: FGF/FGFR2 Signaling Regulates the Generation and Correct Positioning of Bergmann Glia Cells in the Developing Mouse Cerebellum
Source: PLoS One. 2014 Jul 1;9(7):e101124. doi: 10.1371/journal.pone.0101124 (PMC4077754; doi:10.1371/journal.pone.0101124)
Supplement: File S1 — Table S1. Locomotor behaviors of control and Fgfr2 cKO mice. 12 weeks old male Fgfr2lox/lox (control, n = 15) and Nestin-Cre;Fgfr2lox/lox (Fgfr2 cKO, n = 12) mice were tested in the modified hole board (mHB) for horizontal and vertical locomotor abilities. Motor coordination and balance was assessed with the rotating rod apparatus (Rotarod). All values given are mean ± s.e.m. Table S2. Average proportion of Ccnd1+/Pax6− RG/BG precursors/cells among the total number of migrating Ccnd1+ and/or Pax6+ cells in each 50-µm bin in control-, FGF9- or SU5402-treated microexplant cultures. Values represent the average proportion of Ccnd1+/Pax6− RG/BG precursors/cells among the total number of migrating Ccnd1+ and/or Pax6+ cells in each 50-µm bin (distance migrated from the border of the microexplant) and for each treatment, and the 95% confidence interval estimated with a logistic model (in 8 bins and 3 treatments: total cells migrated: 1168, among them RG/BG precursors/cells: 146). Figure S1. Correlation of locomotor and cerebellar phenotypes in adult Fgfr2 cKO mice. (A) Rotarod performance (latencies to fall) of 15 control and 12 Fgfr2 cKO males. Highlighted in red are the control male with the shortest latency to fall (16 sec, ID 30064154), and two Fgfr2 cKO males with the longest (140 sec, ID 30064156) and shortest (25 sec, ID 30064164) latencies to fall. (B–G) Sagittal cerebellar sections from the adult males highlighted in red in (A), counterstained with DAPI (B,D,F) or immunostained for Calb1 (C,E,G). Note the severe cerebellar defects in the ID 30064164 Fgfr2 cKO male with the shortest latency to fall from the Rotarod (F,G). I-X, lobuli of the adult cerebellum. Scale bar (B): 500 µm. Figure S2. The ventral mid-/hindbrain region is not affected in Fgfr2 cKO mice. (A–F) Sagittal views of the ventral MHR from adult control (A,C,E) and Fgfr2 cKO mice (B,D,F), hybridized with riboprobes for Tyrosine hydroxylase (Th; the rate-limiting enzyme for dopamine and noradrenaline s [file pone.0101124.s001.pdf]

FGF/FGFR2 Signaling Regulates the Generation and Correct Positioning of Bergmann Glia Cells in the Developing Mouse Cerebellum

Florian Meier[1*], Florian Giesert[1*], Sabit Delic[1,2*], Theresa Faus-Kessler[1], Friederike Matheus[1], Antonio Simeone[3], Sabine M. Hölter[1], Ralf Kühn[1,4], Daniela M. Vogt Weisenhorn[1,4,5,6], Wolfgang Wurst[1,4,5,6,7§] and Nilima Prakash[1,4§]

Supporting Information

Table S1

Genotype	Horizontal locomotion		Vertical locomotion		Motor coordination (Rotarod)
	Maximum velocity (cm/s)	Total distance travelled (cm)	Latency to first rearing (s)	Number of rearings	Latency to fall (s)
**Control**	64.51±4.03	2627±107	225.1±15.0	2.714±0.624	82.20±13.79
***Fgfr2* cKO**	48.47±2.01	2165±161	295.6±4.4	0.182±0.182	63.33±8.57

Table S2

**Treatment**	**0–50 µm bin**	**50–100 µm bin**	**100–150 µm bin**	**150–200 µm bin**	**200–250 µm bin**	**250–300 µm bin**	**300–350 µm bin**	**350–400 µm bin**
**Control (n=8 explants)**	0.045 [0.03-0.06]	0.037 [0.02-0.06]	0.051 [0.03-0.08]	0.041 [0.02-0.07]	0.026 [0.01-0.05]	0.02 [0.005-0.05]	0.01 [0.001-0.04]	0.000 [0.00-0.00]
**+ FGF9 (n=8 explants)**	0.017 [0.008-0.03]	0.011 [0.003-0.02]	0.013 [0.004-0.03]	0.01 [0.002-0.03]	0.023 [0.01-0.05]	0.000 [0.00-0.00]	0.000 [0.00-0.00]	0.000 [0.00-0.00]
**+ SU5402 (n=6 explants)**	0.019 [0.008-0.04]	0.048 [0.02-0.08]	0.013 [0.002-0.04]	0.038 [0.01-0.08]	0.01 [0.001-0.04]	0.038 [0.006-0.12]	0.058 [0.003-0.3]	0.143 [0.02-0.51]

A

Time spent on rotarod

control		Fgfr2 cKO	
**mouse ID**	**time on rotarod (sec)**	**mouse ID**	**time on rotarod (sec)**
30064150	98	30064149	72
30064151	54	30064152	50
30064153	119	30064155	45
30064154	16	30064156	140
30064158	58	30064157	47
30064159	56	30064162	32
30064160	131	30064163	77
30064161	109	30064164	25
30064166	42	30064165	80
30064168	36	30064167	59
30064170	108	30064169	67
30064171	43	30064174	66
30064172	230		
30064173	51		
30064175	82		

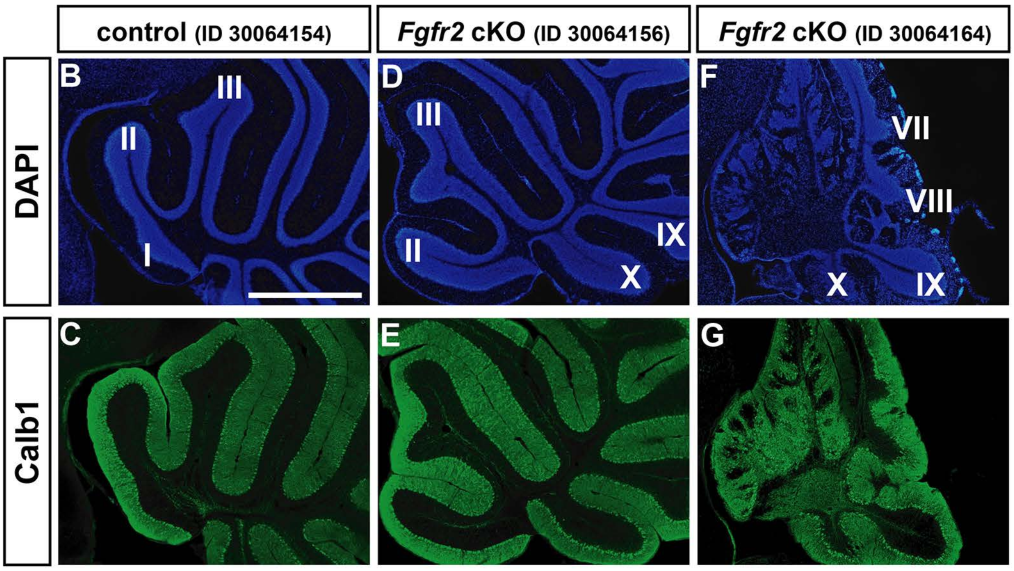

Figure S1

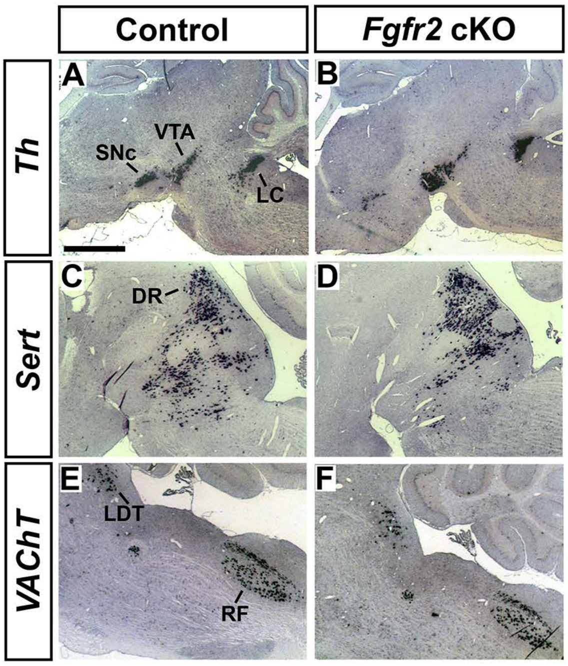

Figure S2

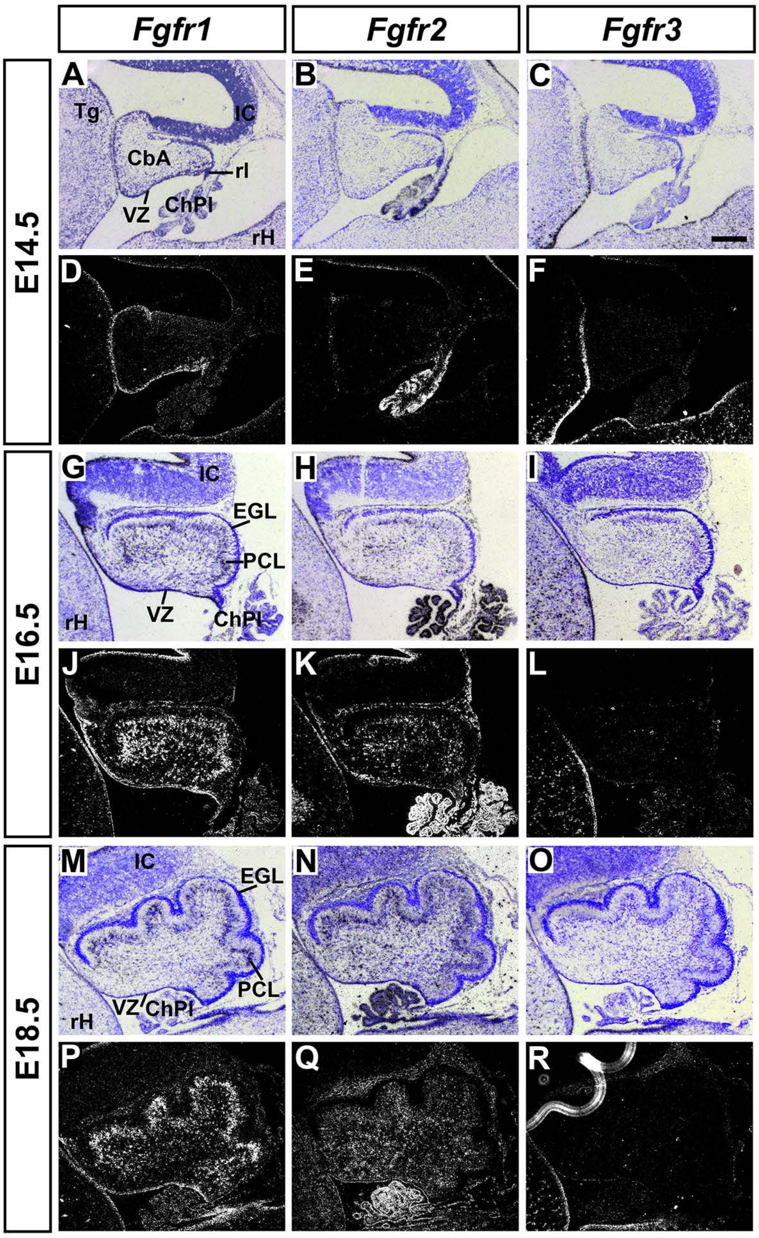
Fgfr1
Fgfr2
Fgfr3
E14.5
E16.5
E18.5
A
B
C
D
E
F
G
H
I
J
K
L
M
N
O
P
Q
R
Tg
IC
CbA
rl
VZ
ChPl
rH
EGL
PCL

Figure S3

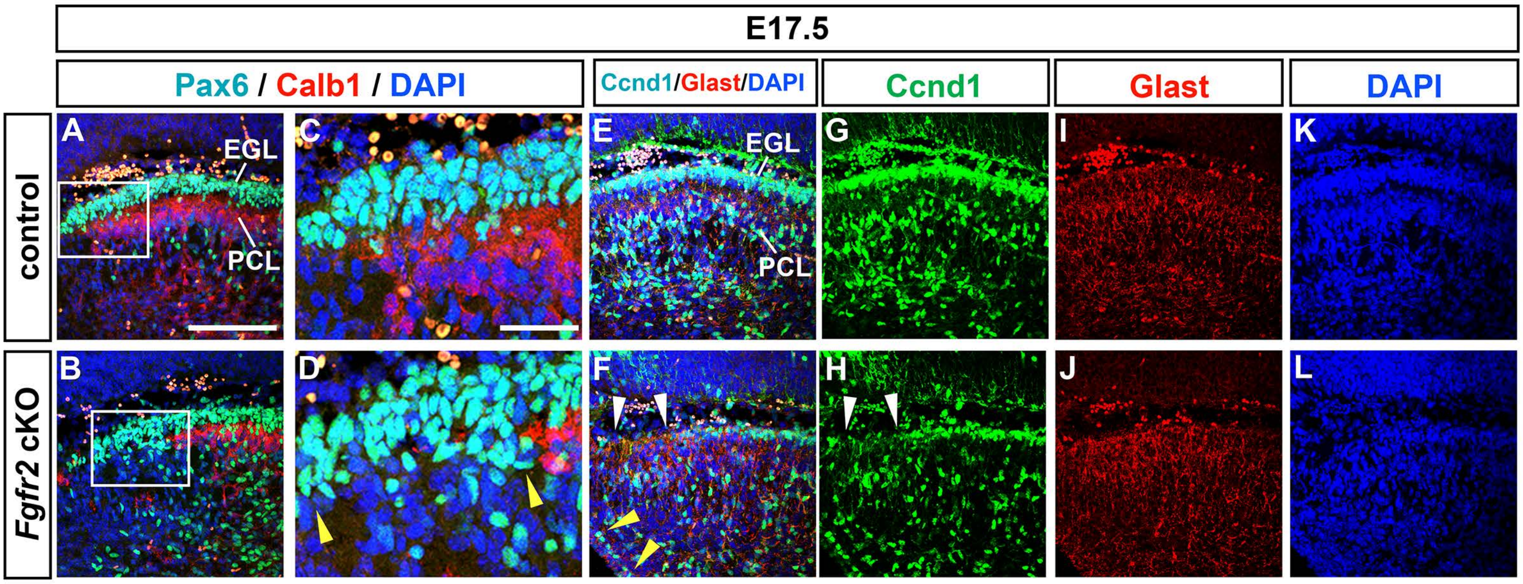
E17.5
Pax6 / Calb1 / DAPI
Ccnd1/Glast/DAPI
Ccnd1
Glast
DAPI
control
Fgfr2 cKO
A
B
C
D
E
F
G
H
I
J
K
L
EGL
PCL
EGL
PCL

Figure S4

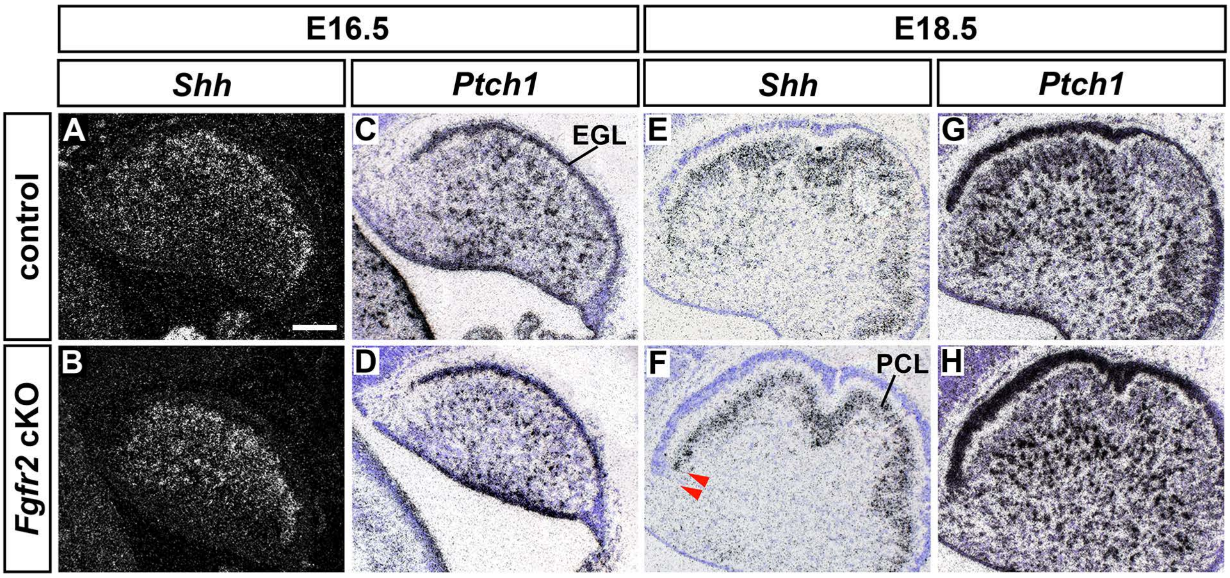
E16.5
E18.5
Shh
Ptch1
Shh
Ptch1
control
Fgfr2 cKO
A
B
C
D
E
F
G
H
EGL
PCL

Figure S5
